# Synthesis of Thionated Perylenediimides: State of the Art and First Investigations of an Alternative to Lawesson’s Reagent †

**DOI:** 10.3390/molecules29112538

**Published:** 2024-05-28

**Authors:** Oksana Kharchenko, Anna Hryniuk, Oksana Krupka, Piétrick Hudhomme

**Affiliations:** 1 Univ Angers, Inserm, CNRS, MINT, SFR ICAT, F-49000 Angers, France; oksana.kharchenko@univ-angers.fr; 2 Univ Angers, CNRS, MOLTECH-Anjou, SFR MATRIX, F-49000 Angers, France; annagrynyuk20@gmail.com

**Keywords:** perylenediimide, thionation, phosphorus pentasulfide

## Abstract

Perylenediimides (PDIs) are composed of a central perylene ring, on which are grafted two imide groups at the peri positions. Thionated PDIs are characterized by the substitution of one or more oxygen atoms of these imide functions with sulfur atoms. This structural modification alters the electronic properties with a redshift of the optical absorption accompanied by modification of the charge transport characteristics compared to their non-thionated counterparts. These properties make them suitable candidates for applications in optoelectronic devices, such as organic light-emitting diodes and organic photovoltaics. Moreover, the presence of sulfur atom(s) can favor the promotion of reactive oxygen species production for photodynamic and photothermal therapies. These thionated PDIs can be synthesized through the post-functionalization of PDIs by using a sulfurizing reagent. Nevertheless, the main drawbacks remain the difficulties in adjusting the degree of thionation and obtaining tri- and tetrathionated PDIs. Up to now, this thionation reaction has been described almost exclusively using Lawesson’s reagent. In the current study, we present our first investigations into an alternative reagent to enhance selectivity and achieve a greater degree of thionation. The association of phosphorus pentasulfide with hexamethyldisiloxane (Curphey’s reagent) clearly demonstrated higher reactivity compared with Lawesson’s reagent to attain multi-thionated PDIs.

## 1. Introduction

**Figure 1:**
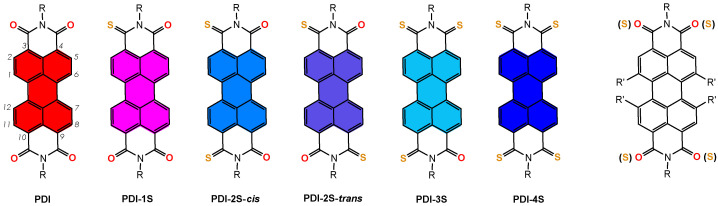
Representation of perylenediimide (PDI) and its thionated derivatives.

Perylenediimides (PDIs) are among the most interesting polycyclic aromatic hydrocarbon structures for chemists, physicists and materials scientists [1,2]. These molecules are composed of a central perylene ring on which are grafted two imide groups at the 3,4 and 9,10 positions (Figure 1). Interest in them continues to grow because they combine thermal and photostability, as well as remarkable optical properties, with a high absorption coefficient and fluorescence quantum yield close to unity. They exhibit strong electron-accepting character with their two electron-withdrawing imide moieties on each side of the perylene backbone and, consequently, they are now considered as one of the best n-type semiconductors, making them ideally suited to applications in organic electronics [3,4], in particular for their use in organic field-effect transistors (OFETs) [5], organic light-emitting diodes (OLEDs) [6] and organic photovoltaic (OPV) [7,8,9,10] devices. However, academic interest has increased in recent years toward the development of new PDI derivatives focusing on biological applications [11,12,13], and their photochemical properties are now widely exploited for developing novel systems for applications in bioimaging, photodynamic therapy (PDT) and photothermal therapy (PTT) [14,15]. Certainly, organic chemistry has played a pivotal role in the development of synthetic strategies for the advancement of such applications [16]. Indeed, it is well-established that optoelectrical properties can be modified significantly by the introduction of substituents in the bay (1, 6, 7 and 12) and ortho (2, 5, 8 and 11) positions. Whereas PDI derivatives were firstly reported in 1913 [17], transformation in the thionated analogs by the substitution of the oxygen atoms of both imide groups with sulfur atoms was only reported and patented almost a century later [18]. It has been demonstrated that the optoelectronic properties depend directly on the degree of thionation and the high potential of these thionated PDIs for specific applications in materials science. In the first part, we present an overview of the methods to synthesize thionated PDIs, showing that this post-functionalization of PDIs has been almost exclusively limited to the use of Lawesson’s reagent to transform imide groups into thioimide groups. In the second part, we describe our initial research into the development of new thionation methods with the aim of obtaining greater selectivity, given that a mixture of monosubstituted **PDI-1S**, disubstituted **PDI-2S-cis** and **PDI-2S-trans**, trisubstituted **PDI-3S** and tetrasubstituted **PDI-4S** is conventionally obtained.

## 2. Overview of the Synthesis of Thionated Perylenediimides

**Figure 2:**
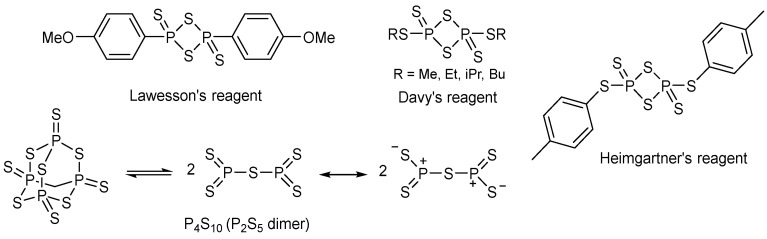
Usual reagents for the thionation of carbonyl groups.

Thionation is a suitable and efficient method for the substitution of an oxygen atom of the carbonyl group with a sulfur atom, using a wide range of thionating reagents such as elemental sulfur (S_8_) [19], hydrogen sulfide [20], phosphorus pentasulfide (P_2_S_5_ or its dimer phosphorus decasulfide P_4_S_10_) [21,22], Lawesson’s reagent (LR) [23,24,25,26], Davy’s reagent [27], Heimgartner’s reagent [28], Curphey’s reagent (P_4_S_10_ with hexamethyldisiloxane HMDSO) [29,30,31], Bergman’s reagent (P_4_S_10_/pyridine) [32,33], Kaushik’s reagent (P_4_S_10_/Al_2_O_3_) [34], Bernthsen’s reagent (S_8_/I_2_) [35], and bis(trimethylsilyl)sulfide or hexamethyldisilathiane (HMDST) (Figure 2) [36].

**Scheme 1:**
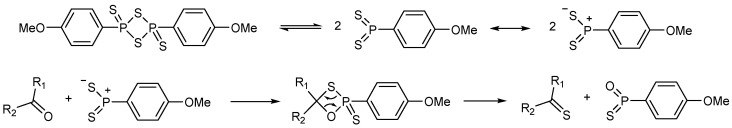
Mechanism of thionation using Lawesson’s reagent.

Among these methods, firstly reported by Lecher et al. in 1956 [37], LR as a phosphorus–sulfur compound has been the most popular for several decades. The LR-mediated thionation reaction is widely used due to its reliability, efficiency, and compatibility with various functional groups. It was successfully applied for the conversion of alcohols, carboxylic acids, ketones, esters and amides. Typically, the thionation reaction involving LR proceeds under mild conditions, usually in an appropriate solvent, often toluene, xylene or dichloromethane. However, LR can release toxic hydrogen sulfide gas upon exposure to water or moisture and is unstable in solution at temperatures above 110 °C with slow decomposition [37,38]. The mechanism of the thionation reaction using LR involves the formation of a highly reactive dithiophosphine ylide that can react with the carbonyl function to form a thiaoxaphosphetane intermediate that evolves into a Wittig-like reaction to give the corresponding thione derivative. This mechanism clearly indicates that each equivalent of LR is capable of delivering two sulfur atoms per reaction (Scheme 1).

As a preamble to the development of the thionation of PDI derivatives, it is important to point out that the thionation reaction of imides has only been described in rare cases [25]. Nevertheless, the first study of the thionation reaction of cyclic imides should be highlighted [39]. Thionation in the phthalimides and naphthtalimides series using LR led to mono- and dithioimides in good yields thanks to the high polarity of carbonyl groups. But, on the other hand, steric hindrance on the nitrogen atom of the imide group was shown to strongly inhibit the replacement of the oxygen atom with a sulfur atom.

The first synthesis of thionated PDI derivatives was patented by A. Fachetti and coll [18]. Thionation was carried out starting from a PDI derivative substituted with (*S*)-1-methylhexyl chains at the imide positions and using LR in 1-methylnaphthalene at 180 °C for 30 min (Table 1, entry 1). Thionated PDI derivatives were separated by silica gel chromatography using toluene as the eluent, from the less polar **PDI-4S** (Rf = 0.92), then **PDI-3S** (Rf = 0.83), **PDI-2S-trans** (Rf = 0.67), **PDI-2S-cis** (Rf = 0.50) to the more polar **PDI-1S** (Rf = 0.23). Optical properties were determined in chloroform, showing a bathochromic shift of the λ_onset_ which increases with the number of sulfur atoms, **PDI-4S** (λ = 765 nm, E_g_ = 1.62 eV), **PDI-3S** (λ = 710 nm, E_g_ = 1.75 eV), **PDI-2S-trans** (λ = 645 nm, E_g_ = 1.92 eV), **PDI-2S-cis** (λ = 645 nm, E_g_ = 1.92 eV), and **PDI-1S** (λ = 605 nm, E_g_ = 2.05 eV), to be compared with PDI starting material (λ = 540 nm, E_g_ = 2.30 eV). Then, this study focused on the production of the **PDI-2S-trans** isomer from PDI derivatives diversely substituted on the imide position using (*R*)-1-methylheptyl (25% yield), racemic 1-methylheptyl (22% yield), 1,3-dimethylbutyl (17% yield), 2-octyldodecyl (25% yield) groups. It should be noted that Davy’s reagent was investigated here as an alternative to the LR yielding **PDI-2S-cis** and **PDI-2S-trans** compounds in 26% and 27% yield, respectively (Table 1, entry 2), or to **PDI-2S-trans** in 22% yield for the 1,6 and 1,7 mixture of dicyanoPDI derivative (Table 1, entry 3) [18].

A few years later, in 2014, D. S. Seferos and coll. nicely synthesized a series of thionated PDIs using LR in refluxing toluene giving from **PDI-1S** to **PDI-3S** in 10–24% yield (Table 1, entry 4). A larger excess of LR and extended reaction time was required to attain **PDI-4S** compound in 29% yield (Table 1, entry 5) [40]. It was noted that the recrystallization of LR from toluene improved yields of the thionation reaction. Additionally, the effect of stoichiometry and the reaction time were studied by C.F.J. Faul and coll. who carried out the synthesis of thionated PDIs in order to investigate the effects of heteroatom substitution in supramolecular polymer systems (Table 1, entry 6) [41]. The best results were obtained using LR in large excess (8 equivalents) and refluxing in toluene for 48 h.

**Figure 3:**
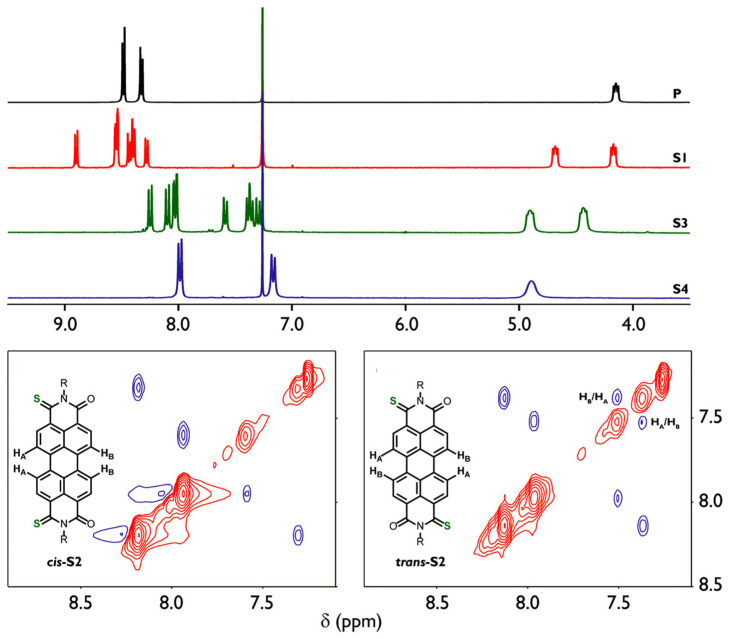
^1^H NMR spectra of the aromatic part of **PDI** (**P**) (R = 3-hexylundecyl chain) and its corresponding thionated derivatives **PDI-1S** (**S1**), **PDI-3S** (**S3**), **PDI-4S** (**S4**), and partial ROESY spectra of **PDI-2S-cis** (**cis-S2**) and **PDI-2S-trans** (**trans-S2**), recorded in CDCl_3_ at 25 °C. Reproduced with permission from reference [40]. Copyright 2014 American Chemical Society.

The degree of thionation can be easily determined by ^1^H NMR spectra (Figure 3), with an exception for **PDI-2S-cis** and **PDI-2S-trans,** for which 2D NMR was required for the assignment of the two isomers [40].

**Figure 4:**
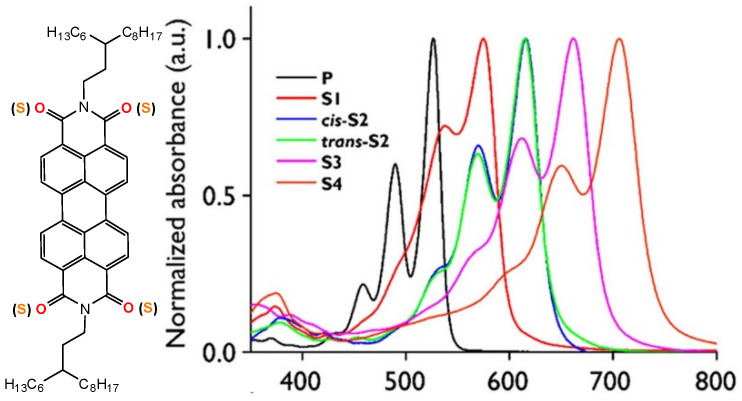
Normalized optical absorption spectra in CHCl_3_ solution of **PDI** (**P**) and its corresponding thionated derivatives **PDI-1S** (**S1**), **PDI-2S-cis** (**cis-S2**), **PDI-2S-trans** (**trans-S2**), **PDI-3S** (**S3**) and **PDI-4S** (**S4**). Reproduced with permission from reference [40]. Copyright 2014 American Chemical Society.

All thionated PDIs are characterized by an absorption maximum ranging from 574 nm for **PDI-1S** to 706 nm for **PDI-4S** (Figure 4, Table 2), but none of them exhibit fluorescence. The presence of a single C=S functional group is sufficient to completely quench PDI emission due to a rapid and highly efficient intersystem crossing (ISC) to a triplet state, this phenomenon being independent of the degree of thionation and attributed to a reordering of the molecular electronic structure.

Both calculated and experimental HOMO/LUMO energies confirmed the slight increase in HOMO energies and the sharp decrease in LUMO energies with increasing sulfur atoms, which justifies the redshift of the maximum absorption (Table 3). Moreover, the influence of the sulfur atoms on the electronic structure and their significant contribution in comparison to the oxygen of diimide groups were achieved.

Furthermore, D.S. Seferos and coll. demonstrated that thionation led to an increase in thin-film transistor electron mobility by two orders of magnitude from **PDI-4O** to **PDI-4S** (0.16 cm^2^·V^−1^·s^−1^) [48]. This synthetic strategy was later expanded in the naphthalenediimide (NDI) series to attain S1 to S4 compounds, replacing the branched 3-hexylundecyl chain by a linear dodecyl chain for studying the influence on solid-state packing [49]. Interestingly, the rate and extent of thionation was increased by heating the reaction mixture more efficiently and at higher temperatures using microwave irradiation to reach thionated NDI derivatives [50].

**Figure 5:**
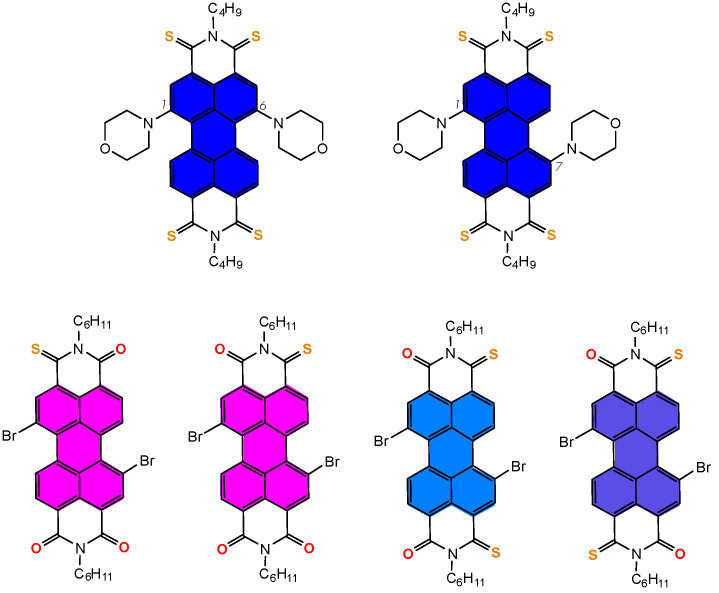
Syntheses of 1,6- and 1,7-isomers of dimorpholino PDI-4S [42], and PDI-1S (two isomers), PDI-2S-cis and PDI-2S-trans from 1,7-dibromoPDI [43].

While the introduction of electron-withdrawing groups into the PDI bay region decreases the energy level of the lowest unoccupied molecular orbital (LUMO), thus increasing the n-type semiconducting character [51,52], the substitution with strong electron-donating groups induces a significant redshift in absorption combined with the fluorescence quenching arising from the electron transfer between the donor groups and the PDI framework. In further developments of thionated PDIs chemistry, N.R. Champness and coll. combined functionalization with electron-donating morpholino groups at the 1,6 and 1,7 bay positions (Table 1, entry 7) and the full thionation of the imide functions to extend absorption in the near infrared (NIR) region (864 nm for the **1,7-PDI-4S** isomer and 838 nm for the **1,6-PDI-4S** isomer in dichloromethane solution (Figure 5) [42]. Moreover, spectroelectrochemical experiments recorded in *o*-dichlorobenzene showed absorption bands at 1568 nm and 1491 nm for the anion-radical species of 1,7-isomer and 1,6-isomer, respectively. These radical anions were more extensively studied by P. Mukhopadhyay and coll. who prepared **PDI-1S** isolated as two isomers and **PDI-2S-cis** and **PDI-2S-trans** (Figure 5) starting from 1,7-dibromoPDI material (Table 1, entry 8) [43]. Remarkably, it was shown that the stability of the radical anion increases with the degree of thionation (the highest 18.8 h for **PDI-2S-trans**), with the vacant *d* orbital of the sulfur atom playing a crucial role in the delocalization of the unpaired electron, thus stabilizing the reduced species.

While these thionated PDI derivatives were designed for use in organic electronics, their synthesis presents notable challenges, resulting in relatively average yields and low selectivity. These factors currently inhibit their large-scale synthesis. More recently, opportunities for their application in PDT and PTT have emerged and aroused growing interest. This is particularly relevant with the very recent progress made in the development of heavy-atom-free photosensitizers (HAF-PSs) because of their potential biocompatibility and prospective applications in PDT [53,54,55,56,57,58]. Indeed, these PDT materials, incorporating sulfur instead of oxygen atoms, are likely to promote ISC, leading to the generation of triplet excitons for reactive oxygen species (ROS) generation. Dithionated PDIs with 1-hexylheptyl imide chains were synthesized by Y. Huang and coll. using LR in refluxing toluene, giving **PDI-2S-cis** and **PDI-2S-trans** in 10.9% and 5.4% yields, respectively (Table 1, entry 9) [44]. Polyethylene glycol-based nanoparticles incorporating these dithionated PDIs were prepared to target tumor tissues via the enhanced permeability retention (EPR) effect. Corresponding **PDI-2S-trans**-based nanoparticles were shown to induce photothermal depression on A549 cells under 660 nm light irradiation both in vitro and in vivo, with a higher photothermal conversion efficiency (PCE) of 58.4% compared to 41.6% for **PDI-2S-cis** based nanoparticles. Furthermore, **PDI-2S-trans** based nanoparticles were shown to generate ROS upon 660 nm laser irradiation, demonstrating an inhibitory effect on tumor growth.

**Figure 6:**
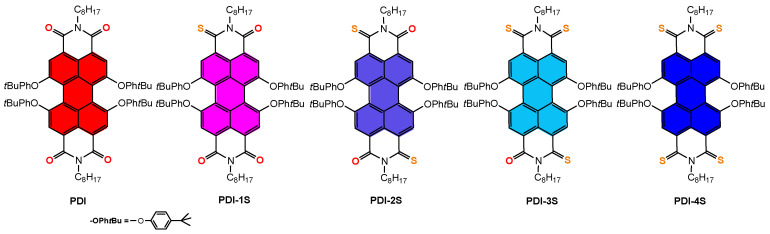
1,6,7,12-tetra-*p-tert*butylphenoxy PDI and corresponding thionated derivatives.

The thionation of the PDI backbone substituted by four 4-*tert*-butylphenoxy groups on the bay region was investigated by M. Yin and coll. (Figure 6) [45]. The synthesis was carried out using LR in xylene under microwave irradiation (Table 1, entry 10).

Using 1,3-diphenylisobenzofuran as a probe to measure singlet oxygen (^1^O_2_) generation and comparing with the photosensitizer methylene blue standard, the ^1^O_2_ quantum yields in these 4-*tert*-butylphenoxy bay-substituted PDIs were shown to gradually decrease with an increase in the thionation degree (Table 4).

**Figure 7:**
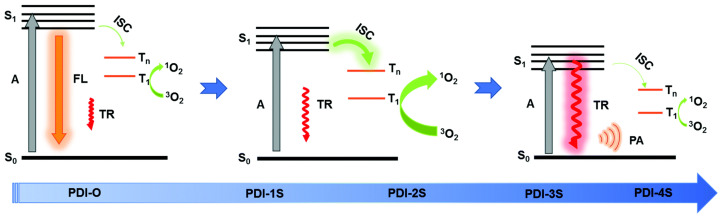
Representation of phototheranostic effects through the degree of PDI thionation (A: absorption, FL: fluorescence, TR: thermal radiation, ISC: intersystem crossing, PA: photoacoustic). Reproduced with permission from reference [45]. Copyright 2021 Royal Society of Chemistry.

This study demonstrated the remarkable capabilities of thionated PDIs in various biomedical applications. By highlighting the influence of the degree of sulfur substitution on ^1^O_2_ generation and photothermal conversion efficiency, as well as the link with the ISC rate constant, the in vitro experiments showed that **PDI-1S**, with its enhanced photodynamic capacity, could be used in tumor phototherapy, while **PDI-4S** might be more suitable as a photothermal and photoacoustic agent in tumor theranostics (Figure 7).

**Figure 8:**
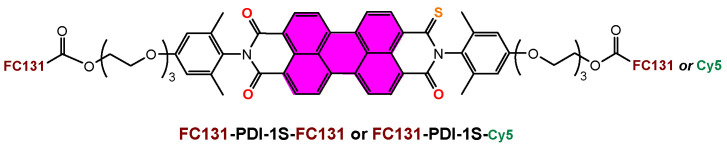
Structures of FC131-PDI-1S-FC131 and FC131-PDI-1S-Cy5 assemblies.

The thionation of PDI substituted with 2-ethylpropyl groups (Table 1, entry 11) and 2,6-dimethylphenyl groups (Table 1, entry 12) on the imide positions was carried out using LR in refluxing toluene by P.T. Chou and coll [46]. It was noted from the last series that there was an increase in the extinction coefficient with the number of sulfur atoms (**PDI-1S**: λ_max_ = 575 nm, ε = 29,200 M^−1^·cm^−1^ in toluene; **PDI-2S-cis**: λ_max_ = 610 nm, ε = 64,300 M^−1^·cm^−1^; **PDI-2S-trans**: λ_max_ = 610 nm, ε = 47,700 M^−1^·cm^−1^; **PDI-3S**: λ_max_ = 660 nm, ε = 66,200 M^−1^·cm^−1^; **PDI-4S**: λ_max_ = 700 nm, ε = 97,700 M^−1^·cm^−1^). In addition, the ability of **PDI-1S** to act as a photosensitiser was exploited with its coupling with FC131 and Cy5 peptides. The key synthetic step was using an LR-mediated thionation in refluxing toluene giving a **PDI-1S** intermediate in 34% yield, which was post-functionalized with the FC131 peptide grafted on each imide position affording the FC131-PDI-1S-FC131 triad (Figure 8). This thionated PDI was also linked, on one imide side, with peptide FC131, and on the other side, with cyanine5 dye, yielding an FC131-PDI-1S-Cy5 assembly. In vitro and in vivo evaluations confirmed the selectivity of these assemblies as active materials in PDT by exhibiting strong two-photon absorption and imaging capabilities of notable anticancer effects, with evidence of exceptional in vivo antitumor efficacy in A549 xenografted tumor mice.

As previously mentioned, the introduction of the less electronegative sulfur atoms on the imide groups leads to a significant extension of the absorption in the long wavelength region. The combination of this phenomenon with an intramolecular charge transfer (ICT) resulting from the introduction of amino groups in the bay position should induce a synergistic shift towards the NIR spectrum. Density Functional Theory (DFT) calculations of the tetrathionated PDI-bearing cyclohexylamino groups in the 1,7 positions showed the high impact of thionation on the LUMO energy level, with a maximum absorption band calculated at 1085 nm (LUMO: −3.72 eV) to be compared with the tetraoxygenated PDI analog presenting an absorption band at 732 nm (LUMO: −3.10 eV), the latter presenting a maximum absorption at 654 nm in dichloromethane solution [59]. Sun and coll. demonstrated this phenomenon with the synthesis of corresponding **PDI-1S** (λ_max_ = 746 nm in DCM), **PDI-2S-trans** (λ_max_ = 795 nm) and **PDI-3S** compounds, the latter exhibiting a remarkable λ_max_ = 854 nm (Table 1, entry 13) [47]. The authors showed ^1^O_2_ generation by these PDIs under 650 nm laser irradiation. In order to solve the problem of solubility in water, silica nanocapsules (SNCs) with encapsulated PDIs were formulated. These **PDI-3S@SNC** exhibited a remarkable power conversion efficiency (PCE) reaching 88% under 808 nm laser irradiation. Additionally, an exceptional photothermal effect under 1064 nm laser irradiation was observed, highlighting its potential as an NIR photothermal agent.

## 3. Investigations of Novel Reagents in Thionated Perylenediimides Synthesis

Lawesson’s reagent (LR) is a widely used tool for the synthesis of thionated compounds, and especially thionated PDIs. Our primary objective was to find an effective alternative for enhancing selectivity and yields for the less available **PDI-3S** and **PDI-4S** compounds. At that point, we decided to investigate this thionation reaction, focusing our research on the phosphorus pentasulfide (P_2_S_5_ or its dimer phosphorus decasulfide P_4_S_10_) reagent. LR and P_4_S_10_ are both commonly used reagents in organic synthesis for converting carbonyl compounds to thiocarbonyl analogs. While both reagents serve a similar purpose, some advantages of using LR over P_4_S_10_ are commonly described. It is often noted that LR proceeds in milder reaction conditions and in a shorter reaction time compared to P_4_S_10_. Moreover, LR is described to provide cleaner reactions with fewer side products compared to P_4_S_10_, leading to higher yields and easier purification of the desired product. Concerning the functional group compatibility, LR is generally more compatible with a wider range of functional groups present in the substrate molecule compared to P_4_S_10_. For instance, LR reagent is less likely to react with sensitive functional groups such as esters and amides. Finally, LR has a relatively milder odor compared to P_4_S_10_, making it more “pleasant” to work with in the laboratory.

The ancestor thionation reagent P_4_S_10_ was first used in 1869 by Henry [60] and Wislicenus [61]. Then, A.W. Hofmann described, in 1878, the transformation of carboxamides into thionoamides, exemplified by the conversion of formanilide into thioformanilide [62]. Due to its low solubility, the reaction is normally carried out with an excess of P_4_S_10_ in refluxing solvent which includes toluene, xylene, dioxan, dimethoxyethane, pyridine and dichloromethane. Furthermore, it was demonstrated that reaction times and reaction temperatures can be reduced significantly when using ultrasound for thionation reaction. Under ultrasound conditions, the use of P_4_S_10_ has been reported to be more selective than LR, producing no reaction side-products [63].

**Figure 9:**
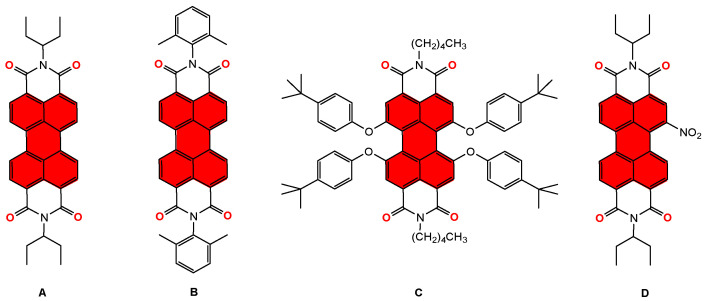
PDI derivatives diversely substituted in the imide and bay positions used in this study.

In this initial investigation of a novel thionation method, several PDI derivatives were selected as starting materials (Figure 9). PDI derivatives **A** and **B** bearing 2-ethylpropyl and 2,6-dimethylphenyl groups as imide substituents were chosen for comparison with thionated compounds obtained using LR and described in the literature. These compounds were prepared by reacting perylene 3,4,9,10-tetracarboxylic dianhydride (PTCDA) with the corresponding amine in imidazole at 150 °C, then purified by column chromatography and precipitated in a mixture of dichloromethane and methanol [64,65]. Compound **C**, prepared as reported in the literature [66], was studied to verify the feasibility of the new procedure, with a hindered PDI tetrasubstituted in the bay region. In addition, this compound can be used to demonstrate the electronic impact of electron-donating tetraphenoxy groups by the mesomeric effect on the reactivity of the thionation reaction. Finally, we proposed PDI **D**, synthesized according to the described procedure [65], bearing a strong electron-withdrawing nitro group in the bay region that is also extremely sensitive to substitution.

**Scheme 2:**
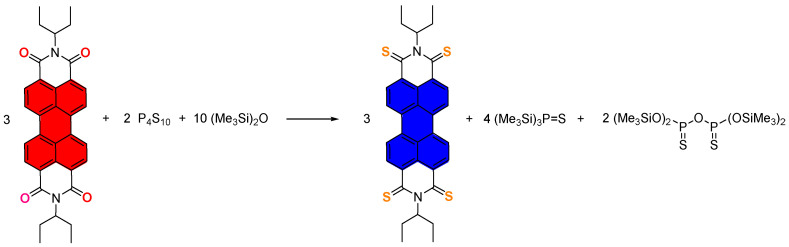
Stoichiometry of the reaction converting PDI A into its corresponding thionated PDI-4S.

Initial attempts using P_4_S_10_ as the sole reagent in refluxing toluene or xylene quickly proved unsuccessful when the reaction was carried out on compound **A**. So, we naturally turned to Curphey’s reagent (CR), which combines P_4_S_10_ and hexamethyldisiloxane (HMDSO). The combination of P_4_S_10_ and HMDSO is described to efficiently convert esters, lactones, lactams and ketones to their corresponding thionated derivatives in yields comparable or superior to those obtained with LR [30]. As our main goal was to optimize the thionation reaction in order to obtain the **PDI-4S** derivative as efficiently as possible, we considered the following stoichiometry presented below (Scheme 2). In this multi-variable optimization study, 0.5 mmol of PDI was employed in anhydrous toluene or xylene (40 mL) at 110 °C or 150 °C, respectively. Consequently, the stoichiometric conditions required 0.33 mmol of P_4_S_10_ and 1.66 mmol of HMDSO. 

The first objective aimed to compare reactions carried out with LR (Table 5, entry 1) or CR (Table 5, entry 2). Under the same reaction conditions, i.e., for 24 h at reflux in toluene, the reaction carried out with LR (6 equiv.) led mainly to the formation of compound **PDI-1S** (35%) alongside small quantities of **PDI-2S-cis** (9%) and **PDI-2S-trans** (8%). The multi-thionated compounds **PDI-3S** and **PDI-4S** were not detected, in agreement with results described for this same compound **A** (Table 1, entry 11) [46]. The first investigation using P_4_S_10_/HMDSO (CR) in the ratio defined above immediately showed that CR significantly accelerated the thionation reaction. The characteristic purple color of the **PDI-1S** compound appeared after about 30 min in refluxing toluene, followed rapidly by a blue color, indicating the formation of multi-thionated compounds. This greater reactivity was confirmed by the absence of starting product **PDI A** at the end of the reaction. Regarding work-up, phosphorus-containing by-products were removed by a mild hydrolysis using a 5.3 M K_2_CO_3_ aqueous solution, according to the literature [31], followed by extraction with chloroform before purification by silica gel chromatography. Elution was carried out using toluene as the eluent affording firstly **PDI-4S** (Rf = 0.96), then **PDI-3S** (Rf = 0.75), **PDI-2S-trans** (Rf = 0.54) and **PDI-2S-cis** (Rf = 0.22), the **PDI-2S-trans** (C_2h_) isomer being less polar than the **PDI-2S-cis** (C_2v_) isomer. The **PDI-1S** derivative (Rf = 0.10 in toluene) was obtained after elution using toluene/EtOAc (95:5) as a mixture of solvents. Whereas **PDI-1S** was isolated in only 4% yield, compounds **PDI-2S-cis** and **PDI-2S-trans** were each obtained in around 40% yield, with at their side **PDI-3S** in 8% yield and **PDI-4S** as traces (Table 5, entry 2). It should be noted that **PDI-2S-gem** was detected by thin-layer chromatography (TLC) but could not be isolated (see TLC in Supporting Information).

After obtaining this initial positive result demonstrating the potential of CR as reagent, the influence of the solvent and temperature reaction was studied. Using xylene at 150 °C under identical stoichiometric conditions, but in a Schlenk flask to take account of the lower boiling point of HMDSO compared to xylene, a significant improvement was observed in the production of the **PDI-3S** compound (17% yield) (Table 5, entry 3). This improvement could also be observed when studying the effect of stoichiometry on the reaction, using a small excess of P_4_S_10_ and HMDSO (Table 5, entry 4) or, more importantly, increasing the quantity of HMDSO (Table 5, entry 5). In the last case, a higher yield (27%) in **PDI-3S** was obtained. However, further increasing the quantity of reagents (Table 5, entry 6) and the reaction time (Table 5, entry 7) quickly revealed certain limitations with a reduction of the overall yield, probably resulting from the degradation process. The successive additions of excess reagent did not significantly improve the reaction efficiency (Table 5, entry 8). Bis(trimethylsilyl)sulfide or hexamethyldisilathiane (HMDST) has been reported as a versatile reagent in the transformation of carbonyl compounds into their thioxo analogs [36]. To our knowledge, reagents P_4_S_10_ and HMDST have never been associated with carrying out a thionation reaction. This reagent was tested under experimental conditions (Table 5, entry 9) allowing for comparison with results obtained using CR (Table 5, entry 4); however, the yields obtained were inconclusive. This initial investigation involving **PDI A** clearly shows the enhanced reactivity of CR compared with LR. It enabled the synthesis of a **PDI-3S** derivative in significant yields, a compound which had not been described until now. Nevertheless, compound **PDI-4S** could only be isolated in trace amounts.

**Figure 10:**
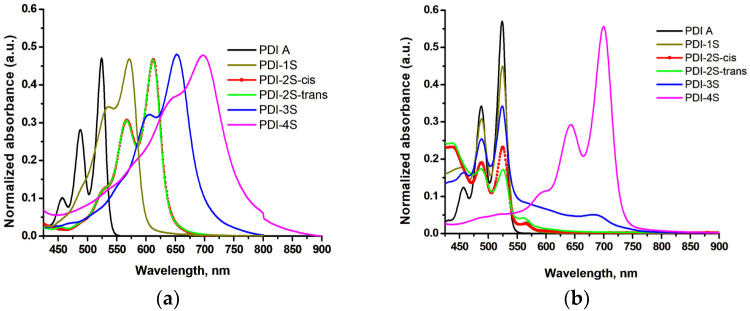
Normalized absorption spectra of PDI A and its thionated PDI derivatives recorded in dichloromethane solution at (**a**) t = 0; (**b**) after 90 h under sunlight in a saturated oxygen atmosphere.

The optical properties of these thionated PDI derivatives were determined in dichloromethane solution, showing the redshift of the maximum absorption with an increase in the number of sulfur atoms (Figure 10a). Corresponding absorption maxima λ_max_ were determined for **PDI A** (525 nm), **PDI-1S** (572 nm), **PDI-2S-cis** (612 nm), **PDI-2S-trans** (612 nm), **PDI-3S** (653 nm), and **PDI-4S** (697 nm).

**Figure 11:**
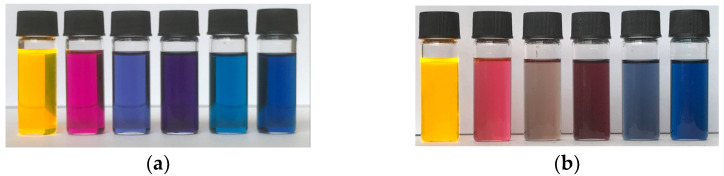

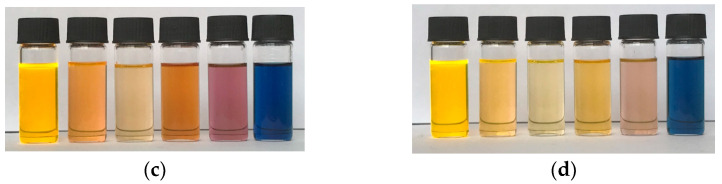
Behavior of PDI A and its thionated derivatives (from left to right: PDI A, PDI-1S, PDI-2S-cis, PDI-2S-trans, PDI-3S, PDI-4S) in dichloromethane under sunlight in a saturated oxygen atmosphere: (**a**) t = 0; (**b**) t = 2 h; (**c**) t = 24 h; (**d**) t = 90 h.

Moreover, we observed that the purple and blue spots on the TLC plate were transformed into orange-red spots, partially regaining their fluorescent properties under light and ambient air conditions. Then, solutions were prepared from starting material **PDI A** and corresponding thionated derivatives from **PDI-1S** to **PDI-4S** in dichloromethane and those saturated with oxygen before sunlight irradiation (Figure 11). We observed the rapid disappearance of the magenta color for **PDI-1S**, along with the blue colors of **PDI-2S-cis** and **PDI-2S-trans** isomers, and also **PDI-3S**. The **PDI-4S** derivative seemed to be much less sensitive to these conditions, resulting in photochemical degradation. This naked eye observation could be paralleled by the measurements of ^1^O_2_ quantum yields described above (Table 4). Mass spectra and UV–visible spectra (Figure 10b) of these samples confirmed the instability in the proposed conditions and the formation of new products, including a return to the **PDI A** starting material resulting from an exchange between the sulfur and oxygen atoms.

The comparison of LR and CR reagents was continued using a PDI **B** compound. The first observation was that the reaction of **PDI B** with LR (Table 5, entry 10) was more efficient than the similar reaction with **PDI A** (Table 5, entry 1). Starting material was quasi-completely converted into thionated PDIs with moderate yields in **PDI-3S** (8%) and **PDI-4S** (5%). These yields were significantly increased as soon as the CR reagent was used, with yields more than doubling in **PDI-3S** (20%) and **PDI-4S** (12%) (Table 5, entry 11). The best results in **PDI-4S** (32%) were obtained using an excess of P_4_S_10_ and HMDSO, under conditions to be compared with those obtained with **PDI A** (Table 5, entry 5). Microwave-assisted thionation of carbonyl compounds using CR was previously described, giving desired products in higher yield and shorter reaction time compared to conventional methods [67,68]. The experiment carried out on **PDI B** under MW irradiation confirmed the importance of solubility (5 × 10^−2^ M for MW experiment instead of 1.25 × 10^−2^ M for experiment reported in Table 5), since only the **PDI-1S** derivative accompanied by unreacted starting material in a large proportion was obtained.

**Scheme 3:**
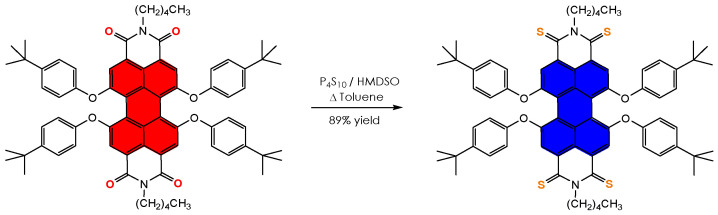
Synthesis of thionated PDI-4S derivative-bearing para *tert*-butylphenoxy groups in the bay positions.

The experimental conditions giving the highest yield in **PDI-4S** for **PDI B** (Table 5, entry 12) were applied to **PDI C** (Table 5, entry 13). Thanks to the improved solubility resulting from the torsion of the PDI backbone, which limits aggregation, and despite the steric hindrance provided by the four para *tert*-butylphenoxy groups in the bay positions, an exceptional reactivity was achieved, leading to **PDI-4S** in an 89% yield (Scheme 3). A slight increase in the quantities of reagents did not improve the yield of the reaction (Table 5, entry 14). As the **PDI-4S** derivative was the only product isolated, the other **PDI-1S**, **PDI-2S** and **PDI-3S** compounds were only detected by mass spectrometry. Once again, this result clearly demonstrates the superiority of CR over LR for obtaining multi-thionated derivatives. The single **PDI-4S** derivative obtained in the example described here should be compared with the mixture containing 24% of a similar derivative obtained using LR in xylene under MW irradiation at 103 °C (Table 1, entry 10).

**Figure 12:**
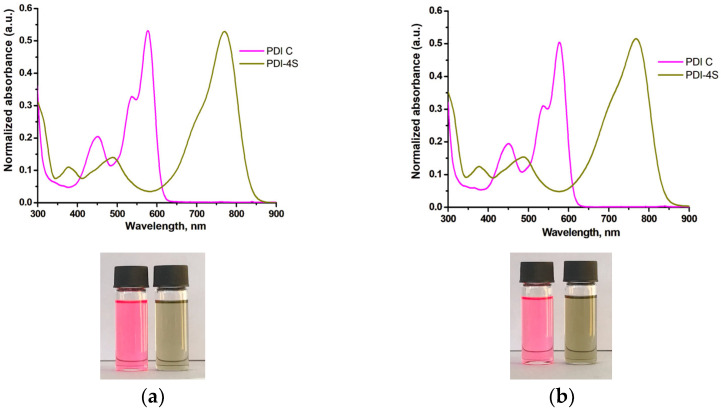
Normalized absorption spectra of PDI C and its PDI-4S derivative recorded in dichloromethane solution. Behavior of PDI C (pink color) and its thionated PDI-4S derivative (grey-green color) at (**a**) t = 0; (**b**) after 90 h under sunlight in a saturated oxygen atmosphere.

The optical properties of PDI **C** and its **PDI-4S** derivative were determined by UV–Vis absorption spectroscopy in dichloromethane solution, confirming the NIR absorption of the **PDI-4S** compound (λ_max_ = 770 nm) compared with tetraoxygenated PDI **C** (λ_max_ = 577 nm). (Figure 12). It can be estimated that each replacement of an oxygen atom of the diimide moieties by a sulfur atom results in a bathochromic shift of approximately 50 nm, whatever the substitution or not on bay region. Solutions of PDI **C** and its **PDI-4S** derivatives in dichloromethane were saturated with oxygen and irradiated with sunlight. We could confirm the good photochemical stability of the **PDI-4S** derivative, as previously noted, for tetrathionated PDI unsubstituted in the bay region.

**Scheme 4:**
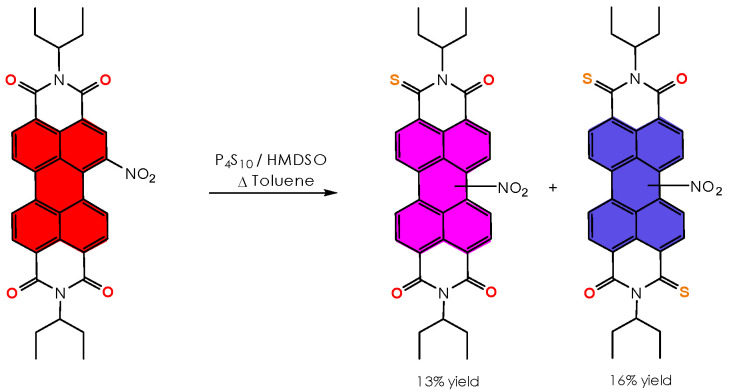
Synthesis of thionated PDI-1S- and PDI-2S-bearing nitro group in bay position.

NitroPDIs are currently attracting significant attention due to their ease of access and higher selectivity of mononitration compared with monobromination [69]. In addition, many types of reactions have been successfully applied to nitroPDIs, ranging from nucleophilic substitution to palladium catalyzed cross-couplings [70,71,72]. The nitro group can also be reduced to an amino group, and further transformation in the corresponding imine using an aldehyde allows azabenzannulated PDI-based materials to be obtained by subsequent photocyclization [73,74]. Consequently, access to thionated PDI derivatives bearing a nitro group in the bay position would open the way to a whole series of post-functionalization processes that are extremely interesting from a synthetic and application point of view. The thionation reaction using CR in refluxing toluene led to a mixture of products, complexed for each compound **PDI-1S**, **PDI-2S cis**, **PDI-2S trans** and **PDI-3S** by the presence of isomers due to the nitro group in the bay position (Scheme 4). The major products isolated were **PDI-1S** and **PDI-2S** compounds, which were characterized by HRMS (Table 5, entry 15). Compound **PDI-3S** was also characterized as a trace by HRMS.

This initial study demonstrates the potential of CR in accessing thionated-PDI derivatives. The impact of the group present at the imide position has an effect on solubility and aggregation [75], and for this thionation reaction, an aromatic substituent seems more favorable than the introduction of a short alkyl chain. On the other hand, the addition of four electron-donating groups in the bay positions, despite the high steric hindrance inherent in this tetrasubstitution, provides a remarkable example of accessing the **PDI-4S** derivative in a practically quantitative yield. Other studies are currently underway to validate the influence of the electronic effect on this thionation reaction. Finally, the presence of a nitro group sensitive to substitution seems compatible with such a reaction. However, achieving selectivity becomes difficult in the case of a monosubstituted derivative in the bay position due to the presence of many possible isomers. This preliminary result seems to indicate that electron-withdrawing groups on the PDI backbone do not favor the thionation reaction. Finally, it is important to highlight that the combination of the P_4_S_10_ reagent with HMDSO would make it possible, for the first time, to synthesize selenated analogues **PDI-1Se** to **PDI-4Se** using the analog P_4_Se_10_ reagent [76]. Whereas remarkable intersystem crossing rates with a complete fluorescence quenching have been observed for thionated PDIs, the theoretical calculations recently reported on selenated PDIs suggest five-order larger ISC rates, signifying high efficiency for photosensitization applications [77].

## 4. Materials and Methods

Experimental procedure for synthesis from PDI derivatives A and B: The reaction and workup should be carried out under an efficient laboratory fume hood. To a solution of PDI (0.5 mmol) in anhydrous toluene or xylene, P_4_S_10_ and HMDSO were added in stoichiometry, as presented in Table 5. The reaction mixture was heated at 110 °C (toluene) or at 150 °C (xylene) under argon atmosphere. After cooling in an ice bath, an aqueous solution of K_2_CO_3_ 5.3 M (1 mL/0.75 mmol P_4_S_10_), then acetone (5 mL), was added. The solution was stirred for 15 min at 0 °C and poured in a separating funnel. Water (100 mL) was added, and the aqueous phase was extracted with chloroform (2 × 100 mL). The organic layer was washed with brine (100 mL), dried over MgSO_4_ and concentrated under vacuum. The crude product was purified by silica gel column chromatography using toluene as the eluent for PDI-4S, PDI-3S, PDI-2S-trans, PDI-2S-cis, and toluene/ethyl acetate (95:5) for PDI-1S.

Experimental procedure for synthesis from PDI-derivative C: To a solution of PDI (561 mg, 0.5 mmol) in anhydrous toluene (40 mL), P_4_S_10_ (334 mg, 0.75 mmol) and HMDSO 1.6 mL (7.5 mmol) were added. The reaction mixture was heated at 110 °C under argon atmosphere for 24 h. After cooling in an ice bath, an aqueous solution of K_2_CO_3_ 5.3 M (1 mL) was added, then acetone (5 mL). The solution was stirred for 15 min at 0 °C and poured in a separating funnel. Water (100 mL) was added, and the aqueous phase was extracted with chloroform (2 × 100 mL). The organic layer was washed with brine (100 mL), dried over MgSO_4_ and concentrated under vacuum. The crude product was purified by silica gel column chromatography using CH_2_Cl_2_/petroleum ether (1/1) as the mixture of eluents. Compound PDI-4S was dissolved in a minimum of CH_2_Cl_2_ and precipitated using MeOH before filtration, giving a green-dark powder (525 mg, 89% yield).

## 5. Conclusions

In conclusion, the current state of the art in the synthesis of thionated perylenediimides (PDIs) clearly shows that the methods used rely almost exclusively on Lawesson’s reagent. However, the reactivity of this reagent seems limited, particularly in terms of access to the multi-thionated derivatives PDI-3S and PDI-4S. Here, we describe a possible alternative using the Curphey’s reagent which combines the P_2_S_5_ reagent (in dimeric form P_4_S_10_) with hexamethyldisiloxane (HMDSO). This initial study clearly demonstrated higher reactivity than Lawesson’s reagent. While direct access to fully thionated PDI-4S appears challenging for PDI derivatives unsubstituted on the perylene backbone, the incorporation of four para *tert*-butylphenoxy substituents in the bay region showed remarkable efficiency with this new synthetic method. In addition, this thionation method seems compatible with the presence of the substitution-sensitive nitro group grafted in the bay position. This preliminary work clearly paves the way for an effective alternative to Lawesson’s reagent in the synthesis of thionated PDIs with possible new developments to access these electron acceptors on a larger scale for applications in organic electronics and biomedicine, especially as metal-free photosensitizers in photodynamic therapy or photothermal therapy.

## Figures and Tables

**Table 1:** Reported experimental conditions and yields in thionated PDI derivatives (Tol.: Toluene; 1-MeNaphth.: 1-Methylnaphthalene; Xyl.: Xylene; *o*-C_6_H_4_Cl_2_: *o*-dichlorobenzene; LR: Lawesson’s reagent; DR: Davy’s reagent; MW = Microwave).

Entry	R	R’	Experimental Conditions	Yield PDI 1S to 4S	Ref
1	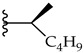	H	LR (2.7 eq.) 1-MeNaphth., 180 °C, 30 min	PDI-1S: < 5% PDI-2S cis: 20–30% PDI-2S trans: 29% PDI-3S: 1% PDI-4S: traces	[18]
2	-CH(CH_2_CH_3_)_2_	H	DR (2 eq.) *o*-C_6_H_4_Cl_2_, 180 °C, 7 min	PDI-2S cis: 26% PDI-2S trans: 27%	[18]
3	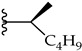	CN (1,6 and 1,7 isomers)	DR (2.2 eq.) *o*-C_6_H_4_Cl_2_, 180 °C, 15 min	PDI-2S trans: 22%	[18]
4	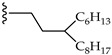	H	LR (2 eq.) Tol., 110 °C, 18 h	PDI-1S: 10% PDI-2S cis: 13% PDI-2S trans: 17% PDI-3S: 24% PDI-4S: traces	[40]
5	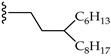	H	LR (5 eq.) Tol., 110 °C, 50 h	PDI-3S: 13% PDI-4S: 29%	[40]
6	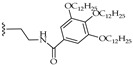	H	LR (8 eq.) Tol., 110 °C, 48 h	PDI-1S: 21% PDI-2S cis: 10% PDI-2S trans: 9%	[41]
7	-C_4_H_9_	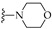 (1,6 and 1,7 isomers)	LR (6 eq.) Tol., 110 °C, 48 h	1,7-PDI-4S: 16% 1,6-PDI-4S: 17%	[42]
8	-C_6_H_11_	Br 1,7 isomer	LR (4 eq.) Tol., 85 °C, 36 h	PDI-1S: 13% and 9% PDI-2S cis: 11% PDI-2S trans: 15%	[43]
9	-CH(C_6_H_13_)_2_	H	LR (10 eq.) Tol., 110 °C, 18 h	PDI-2S cis: 5.4% PDI-2S trans: 10.9%	[44]
10	-C_8_H_17_	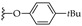 (1,6,7,12 tetra substituted)	LR (4 eq.) Xyl., MW 150 W, 103 °C, 20 min	PDI-1S: 10% PDI-2S trans: 15% PDI-3S: 19% PDI-4S: 24%	[45]
11	-CH(C_2_H_5_)_2_	H	LR (6 eq.) Tol., 110 °C, overnight	PDI-1S: 20% PDI-2S cis: 30% PDI-2S trans: 35%	[46]
12	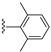	H	LR (6 eq.) Tol., 110 °C, overnight	PDI-1S: 10% PDI-2S cis: 18% PDI-2S trans: 19% PDI-3S: 15% PDI-4S: 9%	[46]
13	-C_6_H_11_	-NH-C_6_H_11_ 1,7 isomer	LR (6 eq.) Tol., 110 °C, 3 days	PDI-1S: 20% PDI-2S-trans: 35% PDI-3S: 12%	[47]

**Table 2:** Optical and electrochemical data of PDI and its thionated derivatives from reference [40].

Compound	λ_max_ (nm)	Optical Band Gap (eV)	E^1/2^_red1_ (V)	E^1/2^_red2_ (V)
PDI (P)	526	2.25	−0.68	−0.91
PDI-1S (S1)	574	2.06	−0.55	−0.72
PDI-2S-cis (S2-cis)	616	1.91	−0.48	−0.57
PDI-2S-trans (S2-trans)	615	1.90	−0.51	−0.61
PDI-3S (S3)	663	1.78	−0.36	−0.45
PDI-4S (S4)	706	1.64	−0.23	−0.33

**Table 3:** Experimental and calculated (in parentheses) HOMO/LUMO energies of thionated derivatives from reference [40]. Copyright 2014 American Chemical Society.

Compound	HOMO (eV)		LUMO (eV)	
PDI (P)	−5.92 (−6.23)	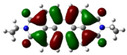	−3.67 (−3.76)	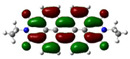
PDI-1S (S1)	−5.85 (−6.15)	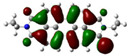	−3.80 (−3.88)	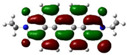
PDI-2S-cis (S2-cis)	−5.78 (−6.09)	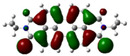	−3.87 (−3.99)	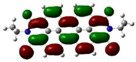
PDI-2S-trans (S2-trans)	−5.74 (−6.08)	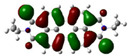	−3.84 (−3.97)	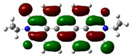
PDI-3S (S3)	−5.77 (−6.04)	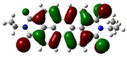	−3.99 (−4.07)	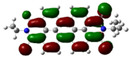
PDI-4S (S4)	−5.76 (−5.98)	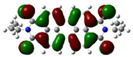	−4.12 (−4.15)	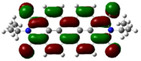

**Table 4:** Optical data and photosensitizing properties of PDI and its thionated derivatives from reference [45]. Measurements were obtained in dichloromethane (DCM), and ^1^O_2_ quantum yields were determined according to methylene blue (Φ_Δ_ = 57% in DCM).

Compound	λ_max_ (nm)	Optical Band Gap (eV)	Φ_PL_ (%)	Φ_Δ_ (%)
PDI	575	2.31	0.92	1
PDI-1S	623	2.13	-	95.6
PDI-2S	666	1.99	-	45.8
PDI-3S	719	1.81	-	11.1
PDI-4S	769	1.68	-	0.5

**Table 5:** Experimental results from reactions carried out using 0.5 mmol of PDI **A**, **B**, **C** or **D** in toluene (Tol.) at 110 °C or xylene (Xyl.) at 150 °C. (nd = not detected).

Entry	PDI	Reagent	Solvent, Time	Yields (%)					
				PDI	PDI-1S	PDI-2S-cis	PDI-2S-trans	PDI-3S	PDI-4S
1	A	LR (3 mmol)	Δ Tol., 24 h	21	35	9	8	nd	nd
2	A	P_4_S_10_ (0.33 mmol) HMDSO (1.66 mmol)	Δ Tol., 24 h	trace	4	40	42	8	trace
3	A	P_4_S_10_ (0.33 mmol) HMDSO (1.66 mmol)	Δ Xyl., 24 h	nd	5	33	31	17	trace
4	A	P_4_S_10_ (0.75 mmol) HMDSO (3.75 mmol)	Δ Tol., 24 h	nd	trace	28	29	20	trace
5	A	P_4_S_10_ (0.75 mmol) HMDSO (7.5 mmol)	Δ Tol., 24 h	nd	1	24	23	27	trace
6	A	P_4_S_10_ (1.5 mmol) HMDSO (15 mmol)	Δ Tol., 24 h	nd	nd	12	15	7	trace
7	A	P_4_S_10_ (1.5 mmol) HMDSO (15 mmol)	Δ Tol., 120 h	nd	nd	11	18	9	trace
8	A	P_4_S_10_ (3 × 0.33 mmol) HMDSO (3 × 1.66 mmol)	Δ Tol., 3 × 24 h	nd	2	27	28	16	trace
9	A	P_4_S_10_ (0.75 mmol) HMDST (3.75 mmol))	Δ Tol., 24 h	2	13	18	23	1	trace
10	B	LR (3 mmol)	Δ Tol., 24 h	trace	13	19	15	8	5
11	B	P_4_S_10_ (0.33 mmol) HMDSO (1.66 mmol)	Δ Tol., 24 h	nd	11	14	17	20	12
12	B	P_4_S_10_ (0.75 mmol) HMDSO (7.5 mmol)	Δ Tol., 24 h	nd	trace	2	2	18	32
13	C	P_4_S_10_ (0.75 mmol) HMDSO (7.5 mmol)	Δ Tol., 24 h	nd	trace	trace	trace	trace	89
14	C	P_4_S_10_ (1 mmol) HMDSO (10 mmol)	Δ Tol., 24 h	nd	trace	trace	trace	trace	84
15	D	P_4_S_10_ (0.75 mmol) HMDSO (7.5 mmol)	Δ Tol., 24 h	nd	13	16		trace	nd

## Data Availability

Not applicable.

## Supplementary Materials

The following supporting information can be downloaded at: https://www.mdpi.com/article/10.3390/molecules29112538/s1. Figures S1–S22: ^1^H, ^13^C and HRMS spectra of thionated perylenediimides; Figures S23–S24: Photochemical degradation study.
