# Supplementary material for: Synthesis of Thionated Perylenediimides: State of the Art and First Investigations of an Alternative to Lawesson’s Reagent
Source: Molecules. 2024 May 28;29(11):2538. doi: 10.3390/molecules29112538 (PMC11173947; doi:10.3390/molecules29112538)
Supplement: Supplementary file 1 [file molecules-29-02538-s001.zip › molecules-3014404-supplementary.pdf]

Thionated Perylenediimides: State of the Art and First Investigations of an Alternative to the Lawesson's Reagent

Oksana Kharchenko,[1] Anna Hryniuk,[2] Oksana Krupka,[1*] and Piétrick Hudhomme[2*]

[1] Univ Angers, Inserm, CNRS, MINT, SFR ICAT, F-49000 Angers, France
[2] Univ Angers, CNRS, MOLTECH-Anjou, SFR MATRIX, F-49000 Angers, France

CAPTIONS and LEGENDS

Materials and Methods

Experimental Procedures

Figure S1. HRMS spectrum of **PDI A 1S**
Figure S2. HRMS spectrum of **PDI A 2S-cis**
Figure S3. HRMS spectrum of **PDI A 2S-trans**
Figure S4. ^{1}H spectrum of **PDI A 3S**
Figure S5. ^{13}C spectrum of **PDI A 3S**
Figure S6. HRMS spectrum of **PDI A 3S**
Figure S7. Aromatic part of the ^{1}H spectrum of **PDI A 4S**
Figure S8. HRMS spectrum of **PDI A 4S**
Figure S9. HRMS spectrum of **PDI B 1S**
Figure S10. HRMS spectrum of **PDI B 2S-cis**
Figure S11. HRMS spectrum of **PDI B 2S-trans**
Figure S12. HRMS spectrum of **PDI B 3S**
Figure S13. HRMS spectrum of **PDI B 4S**
Figure S14. ^{1}H spectrum of **PDI C 4S**
Figure S15. ^{13}C spectrum of **PDI C 4S**
Figure S16. HRMS spectrum of **PDI C 4S**
Figure S17. HRMS spectrum of a mixture of **PDI C 1S + PDI C 2S + PDI C 3S**
Figure S18. ^{1}H spectrum of **PDI D 1S**
Figure S19. HRMS spectrum of **PDI D 1S**
Figure S20. ^{1}H spectrum of **PDI D 2S**
Figure S21. HRMS spectrum of **PDI D 2S**
Figure S22. HRMS spectrum of **PDI D 3S**
Figure S23. Evolution of photochemical degradation of **PDI A** and its thionated derivatives under sunlight irradiation in oxygen saturated solution
Figure S24. MS spectra of **PDI A 1S** and **PDI A 2S-cis** from solutions in CH_2Cl_2 saturated with oxygen after sunlight irradiation

I. Materials and Methods

Chemicals were purchased from Sigma-Aldrich (Lawesson's reagent, P_4S_{10}, HMDSO, HMDST), Thermo Scientific (K_2CO_3).

Solvents were purchased from Fisher Chemical (Toluene HPLC grade, MeOH HPLC grade, Petroleum ether, Ethyl acetate, Toluene 99% for chromatography), Carlo Erba (Xylene, $CHCl_3$, CH_2Cl_2), VWR Chemicals (Acetone), Sigma-Aldrich ($CDCl_3$). Toluene and Xylene were dried over Na/benzophenone.

Perylenediimide-based starting materials **A**,[1] **B**,[2] **C**,[3] **D**[1] were prepared according to literature.

Thin-layer chromatography (TLC) was conducted on pre-coated aluminum sheets with 0.20 mm Merck Alugram SIL G/UV254 with fluorescent indicator UV254. Column chromatography was carried out using Sigma-Aldrich silica gel 60 (particle size 63-200 μm).

Nuclear magnetic resonance (NMR) ^{1}H and ^{13}C spectra were obtained on a Bruker 600 MHz spectrometer (600 MHz for ^{1}H, 151 MHz for ^{13}C). or Bruker 500 MHz spectrometer (500 MHz for ^{1}H, 125 MHz for ^{13}C). Chemical shifts were reported in ppm according to tetramethylsilane using the solvent residual signal as an internal reference ($CDCl_3$: ^{1}H = 7.26 ppm, ^{13}C = 77.16 ppm or toluene-d8 ^{1}H = 6.97 ppm). Coupling constants (J) were given in Hz. Resonance multiplicity was described as s (singlet), d (doublet), t (triplet), quint. (quintet) d.quint (doublet of quintet), m (multiplet).

MALDI-TOF spectra were performed on a Bruker Daltonics Biflex III (SFR Matrix, MOLTECH-Anjou, Angers) using DCTB (trans-2-[3-(4-tert-butylphenyl)-2-methyl-2-propenylidene]malononitrile) as matrix. High resolution mass spectrometry (HRMS) was performed with a JEOL JMS-700 B/E.

UV-Vis absorption spectra were recorded on a Shimadzu UV-1800 UV-Vis spectrophotometer using quartz cell (pathlength of 1 cm).

II. Experimental Procedures

Compound A:

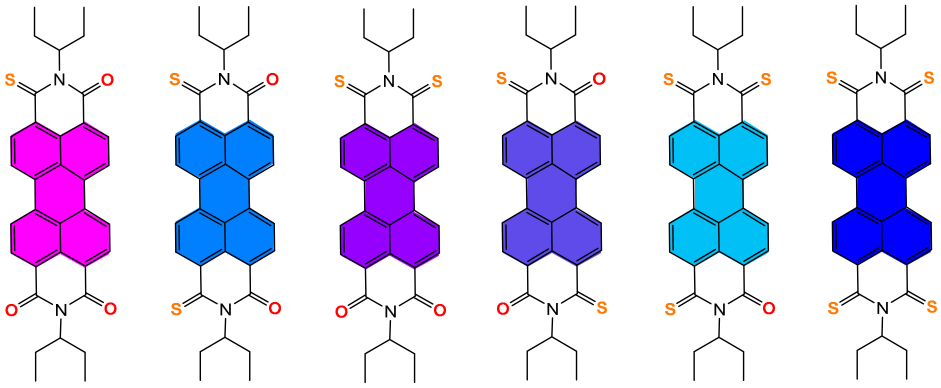

PDI-1S PDI-2S-*cis* PDI-2S-*gem* PDI-2S-*trans* PDI-3S PDI-4S

The reaction and work-up should be carried out under an efficient laboratory fume hood.

To a solution of PDI (0.5 mmol) in anhydrous toluene or xylene (40 mL) was added P_4S_{10} and HMDSO in stoichiometry presented in Table 5 of the manuscript. The reaction mixture was heated at 110°C (toluene) or at 150°C (xylene) under argon atmosphere. After cooling in an ice-bath, an aqueous solution of K_2CO_3 5.3 M (1 mL / 0.75 mmol P_4S_{10}) was added, then acetone (5 mL). The solution was stirred for 15 min at 0°C and poured in a separating funnel. Water (100 mL) was added and the aqueous phase was extracted with chloroform (2x100 mL). The organic layer was washed with brine (100 mL), dried over $MgSO_4$ and concentrated under vacuum. The crude product was purified by silica gel column chromatography using toluene as the eluent for PDI-4S, PDI-3S, PDI-2S-trans, PDI-2S-cis then toluene/EtOAc (95:5) for PDI-1S. All compounds were dissolved in minimum of CH_2Cl_2 and precipitated using MeOH before filtration.

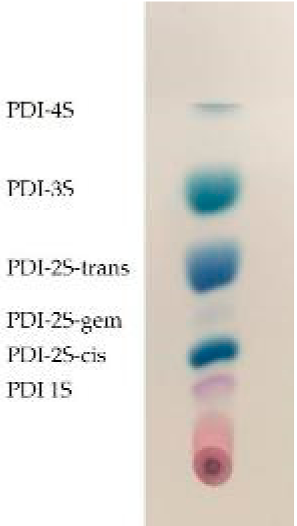

Thin-layer chromatography of the crude reaction after extraction and before purification on silica gel (eluent: toluene 100%)

PDI-1S:

^{1}H NMR and ^{13}C NMR spectra were previously reported in literature.[4]

HRMS (MALDI-TOF, DCTB pos. mode, M-H^{+}) m/z: Calcd for $C_{34}H_{29}N_2O_3S$: 545.18934; Found: 545.1888 (error : 0.95 ppm)

PDI-2S-cis:

^{1}H NMR and ^{13}C NMR spectra were previously reported in literature.[4]

HRMS (MALDI-TOF, DCTB neg mode) m/z: Calcd for $C_{34}H_{30}N_2O_2S_2$: 562.17542; Found: 562.1766 (error 2.06 ppm).

PDI-2S-trans:

^{1}H NMR and ^{13}C NMR spectra were previously reported in literature.[4]

HRMS (MALDI-TOF, DCTB neg. mode) m/z: Calcd for $C_{34}H_{30}N_2O_2S_2$: 562.17542; Found: 562.1756 (error : 0.36 ppm)

PDI-3S:

^{1}H NMR (600 MHz, $CDCl_3$) δ: 9.05 (m, 1H), 8.92 (m, 2H), 8.64 (d, ^{3}J = 8.0 Hz, 1H), 8.56 (d, ^{3}J = 8.0 Hz, 1H), 8.46 – 8.41 (m, 3H), 6.68 (quint, ^{3}J = 7.7 Hz, 1H), 6.31 (quint, ^{3}J = 7.7 Hz, 1H), 2.58 (d.quint, ^{3}J = 7.6 Hz and ^{2}J = 14.8 Hz, 2H), 2.33 (d.quint, ^{3}J = 7.6 Hz and ^{2}J = 14.8 Hz, 2H), 2.21 (d.quint, ^{3}J = 7.6 Hz and ^{2}J = 14.8 Hz, 2H), 2.07 (d.quint, ^{3}J = 7.6 Hz and ^{2}J = 14.8 Hz, 2H), 0.97 (t, ^{3}J = 8 Hz, 6H), 0.95 (t, ^{3}J = 8 Hz, 6H).

^{13}C NMR (151 MHz, $CDCl_3$) δ: 196.5, 160.9, 138.6, 138.3, 136.4, 134.6, 133.9, 133.2, 131.9, 129.2, 128.4, 128.0, 127.7, 126.0, 125.9, 125.4, 124.4, 124.1, 123.7, 123.6, 123.3, 70.8, 65.6, 25.6, 25.2, 11.45, 11.41.

HRMS (MALDI-TOF, DCTB neg. mode) m/z: Calcd for $C_{34}H_{30}N_2OS_3$: 578.15257; Found: 578.1516 (error: 1.76 ppm)

PDI-4S:

This compound was obtained as traces and not in sufficient quantity to record the NMR spectrum in $CDCl_3$. However, the PDI signals were shown in the spectrum recorded in toluene-d8 at 100°C.

^{1}H NMR (500 MHz, Toluene-d8) δ: 8.86 (d, ^{3}J = 8 Hz), 7.83 (d, ^{3}J = 8 Hz)

HRMS (MALDI-TOF, DCTB neg. mode) m/z: Calcd for $C_{34}H_{31}N_2S_4$: 594.12973; Found: 594.1303 (error : 1.01 ppm)

Compound B:

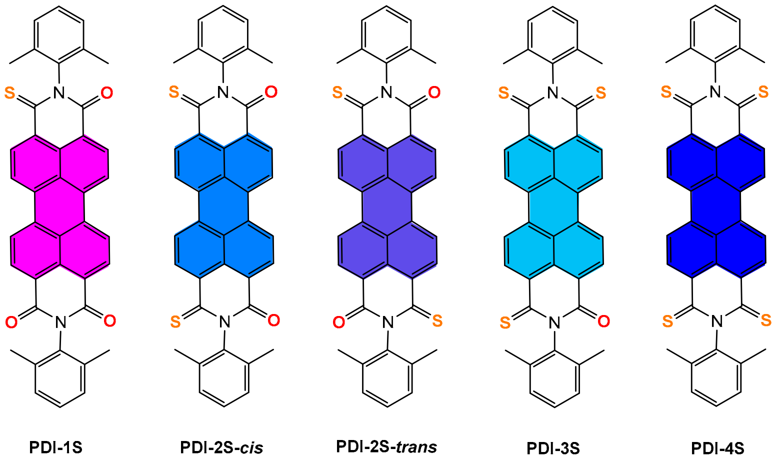

PDI-1S:

^{1}H NMR and ^{13}C NMR spectra were previously reported in literature.[4]

HRMS (MALDI-TOF, DCTB neg. mode) m/z: Calcd for $C_{40}H_{26}N_2O_3S$: 614.16696; Found: 614.1678 (error: 1.43 ppm)

PDI-2S-cis:

^{1}H NMR and ^{13}C NMR spectra were previously reported in literature.[4]

HRMS (MALDI-TOF, DCTB pos. mode, M+H$^+$) m/z: Calcd for $C_{40}H_{27}N_2O_2S_2$: 631.15085; Found: 631.1516 (error: 1.14 ppm)

PDI-2S-trans:

^{1}H NMR and ^{13}C NMR spectra were previously reported in literature.[4]

HRMS (MALDI-TOF, DCTB pos. mode, M+H$^+$) m/z: Calcd for $C_{40}H_{27}N_2O_2S_2$: 631.15085; Found: 631.1511 (error : 0.35 ppm)

PDI-3S:

^{1}H NMR and ^{13}C NMR spectra were previously reported in literature.[4]

HRMS (MALDI-TOF, DCTB neg. mode) m/z: Calcd for $C_{40}H_{26}N_2OS_3$: 646.12127; Found: 646.1205 (error: 1.18 ppm)

PDI-4S:

^{1}H NMR and ^{13}C NMR spectra were previously reported in literature.[4]

HRMS (MALDI-TOF, DCTB pos. mode) m/z: Calcd for $C_{40}H_{26}N_2S_4$: 662.09733; Found: 662.0960 (2.08 ppm)

Compound C:

The reaction and work-up should be carried out under an efficient laboratory fume hood.

To a solution of PDI (561 mg, 0.5 mmol) in anhydrous toluene (40 mL) was added P_4S_{10} (334 mg, 0.75 mmol) and HMDSO 1.6 mL (7.5 mmol). The reaction mixture was heated at 110°C under argon atmosphere for 24h. After cooling in an ice-bath, an aqueous solution of K_2CO_3 5.3 M (1 mL) was added, then acetone (5 mL). The solution was stirred for 15 min at 0°C and poured in a separating funnel. Water (100 mL) was added and the aqueous phase was extracted with chloroform (2x100 mL). The organic layer was washed with brine (100 mL), dried over $MgSO_4$ and concentrated in vacuo. The crude product was purified by silica gel column chromatography using CH_2Cl_2/petroleum ether (1/1) as the mixture of eluents. Compound PDI-4S was dissolved in minimum of CH_2Cl_2 and precipitated using MeOH before filtration giving a green-dark powder (525 mg, 89% yield).

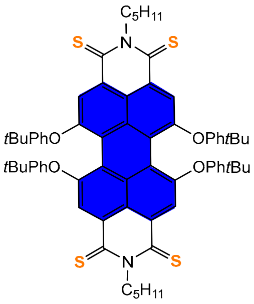

PDI-4S

PDI-4S:

^{1}H NMR (600 MHz, $CDCl_3$) δ: 8.50 (s, 4H), 7.25 (d, ^3J = 8.2 Hz, 8H), 6.82 (d, ^3J = 8.2 Hz, 8H), 5.24 (t, ^3J = 8 Hz, 4H), 1.84 (quint., ^3J = 8 Hz, 4H), 1.38-1.34 (m, 8H), 1.31 (s, 36H), 0.90 (t, ^3J = 7 Hz, 6H).

^{13}C NMR (151 MHz, $CDCl_3$) δ: 188.5, 156.1, 153.2, 147.2, 132.2, 129.3, 126.7, 125.9, 120.9, 119.1, 115.3, 34.5, 34.4, 31.6, 28.9, 25.2, 22.4, 14.2.

HRMS (MALDI-TOF, DCTB neg. mode) m/z: Calcd for $C_{74}H_{78}N_2O_4S_4$: 1186.48499; Found: 1186.4870 (error: 1.73 ppm)

Compound D:

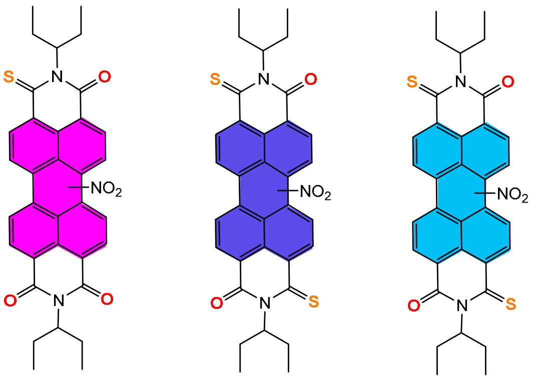

PDI-1S:

HRMS (MALDI-TOF, DCTB neg. mode) m/z: Calcd for $C_{34}H_{29}N_3O_5S$: 591.18334; Found: 591.1841 (error: 1.34 ppm)

PDI-2S:

HRMS (MALDI-TOF, DCTB neg. mode) m/z: Calcd for $C_{34}H_{29}N_3O_4S_2$: 607.16050; Found: 607.1614 (error: 1.49 ppm)

PDI-3S:

HRMS (MALDI-TOF, DCTB neg. mode) m/z: Calcd for $C_{34}H_{29}N_3O_3S_3$: 623.13765; Found: 623.1386 (error: 1.56 ppm)

PDI A 1S:

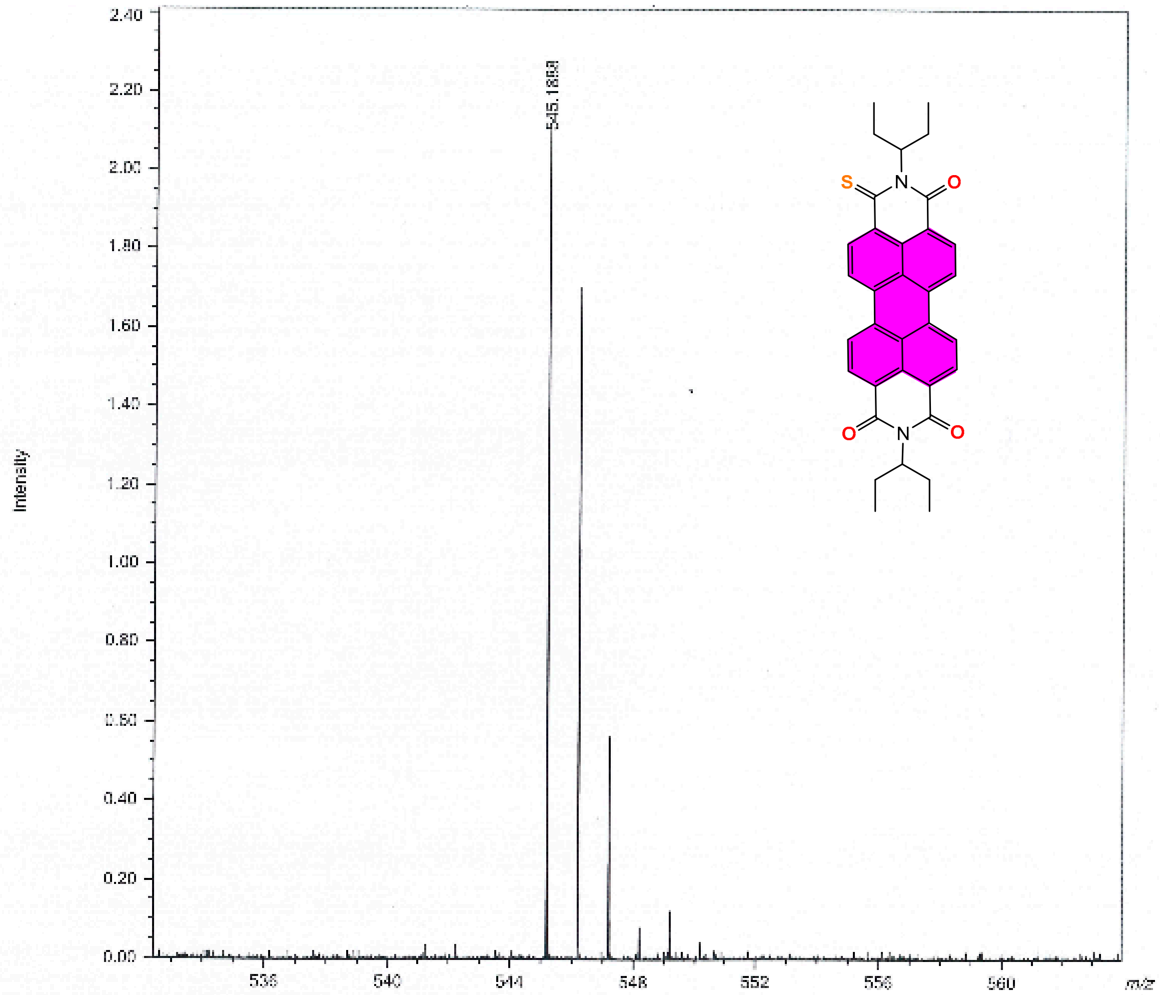

Elemental Composition Estimation

Parameters:

Mass	Tolerance	Electron Mode	Charge	DBE Range	Max Results
545.18882 ± 0.00164	3.0 ppm	Even	+1	-0.5 - 200.0	100

Elements

C 0 - 50 H 0 - 200 N 0 - 5 O 0 - 5 Br 0 - 0 S 1 - 1

Results:

#	Formula	Mass	DBE	Abs. Error (u)	Error (u)	Error (ppm)
1	C34 H20 N2 O3 S	545.18934	21.5	0.00052	-0.00052	-0.95

Figure S1. HRMS spectrum of **PDI A 1S**

PDI A 2S-cis:

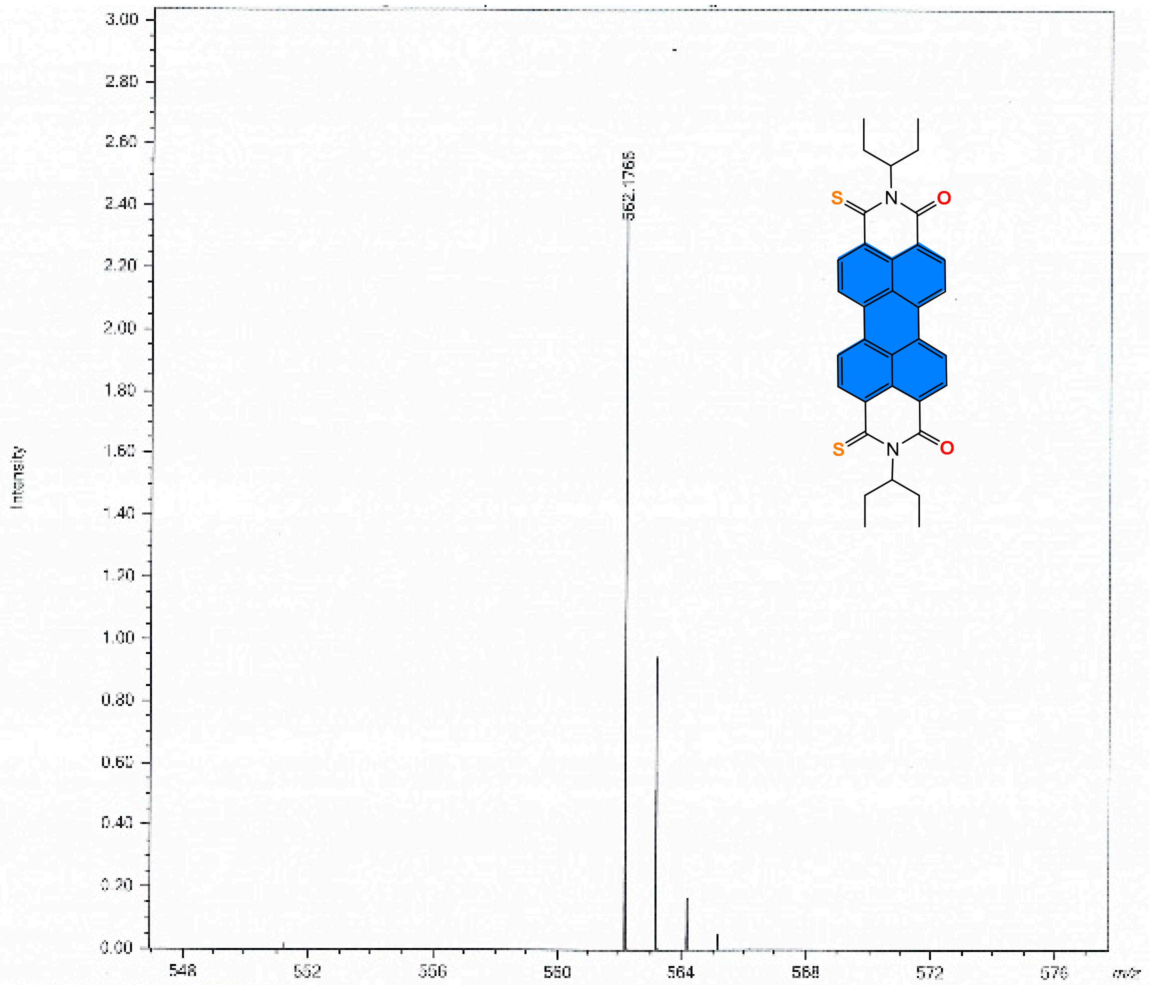

Elemental Composition Estimation

Parameters:

Mass	Tolerance	Electron Mode	Charge	DBE Range	Max Results
562.17658 ± 0.00141	2.5 ppm	Odd	1	-0.5 - 50.0	100

Elements

C 0 - 100 H 0 - 50 N 1 - 5 O 0 - 5 Br 0 - 0 S 1 - 5

Results:

#	Formula	Mass	DBE	Abs. Error (u)	Error (u)	Error (ppm)
1	C34 H30 N2 O2 S2	562.17542	21.0	0.00116	0.00116	2.06

Figure S2. HRMS spectrum of **PDI A 2S-cis**

PDI A 2S-trans:

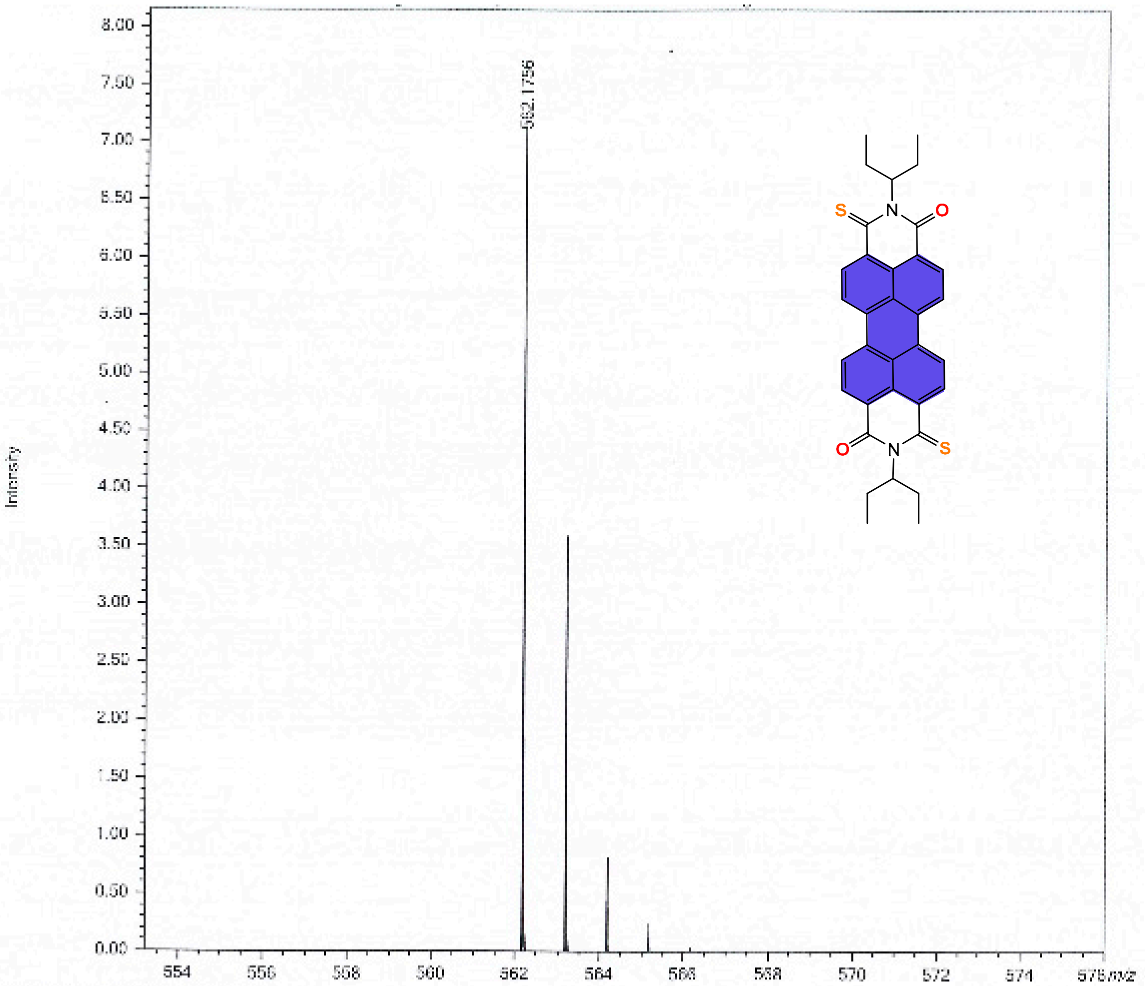

Elemental Composition Estimation

Parameters:

Mass	Tolerance	Electron Mode	Charge	DBE Range	Max Results
562.17562 ± 0.00056	1.0 ppm	Odd	1	-0.5 - 50.0	100

Elements

C 0 - 100 H 0 - 50 N 1 - 5 O 0 - 5 Br 0 - 0 S 1 - 5

Results:

#	Formula	Mass	DBE	Abs. Error (u)	Error (u)	Error (ppm)
1	C34 H30 N2 O2 S2	562.17542	21.0	0.00020	0.00020	0.36

Figure S3. HRMS spectrum of **PDI A 2S-trans**

PDI A 3S:

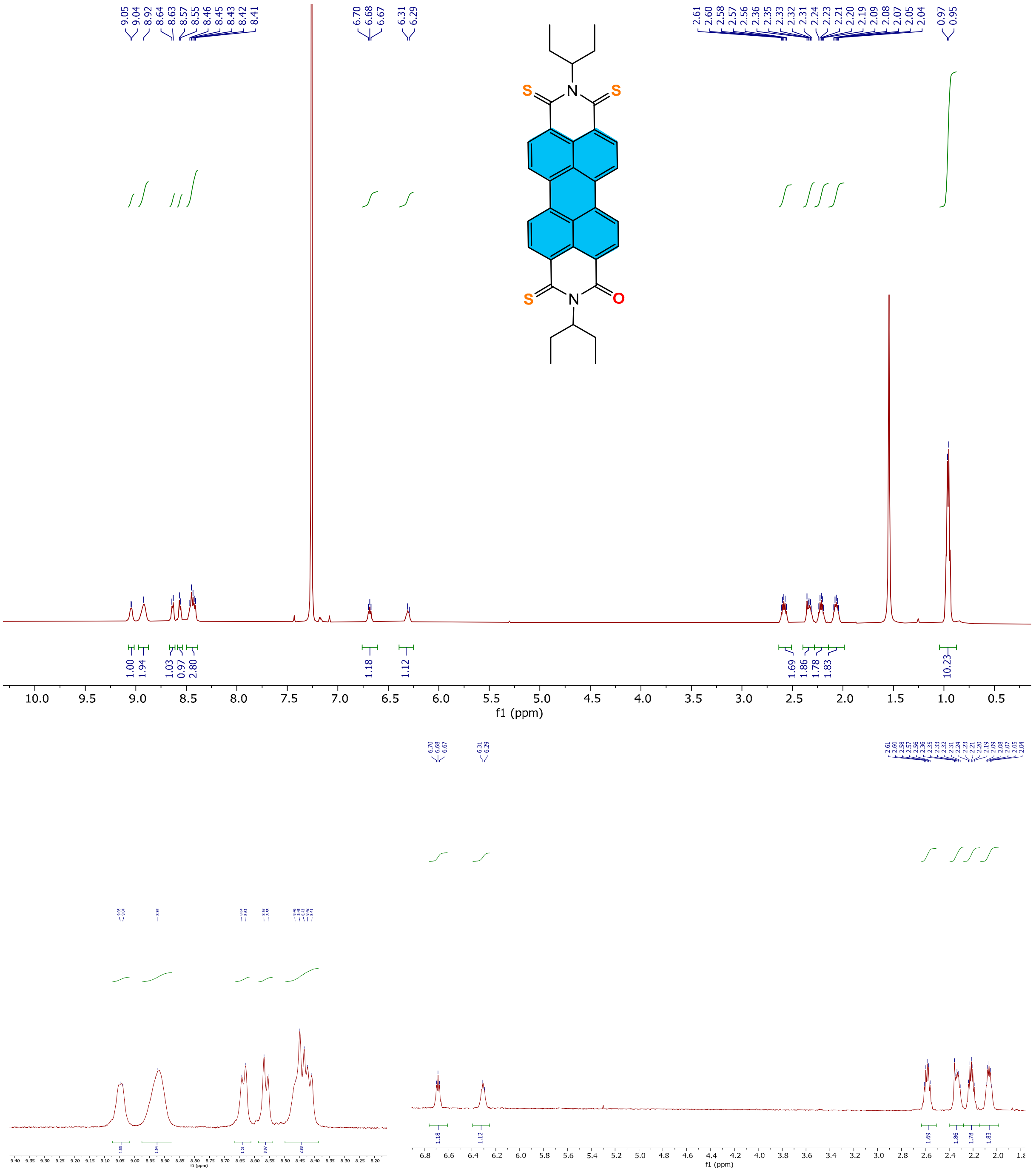

Figure S4. ^{1}H spectrum of **PDI A 3S** recorded in $CDCl_3$ (top) and enlargement of the aromatic part (left) and aliphatic part (right)

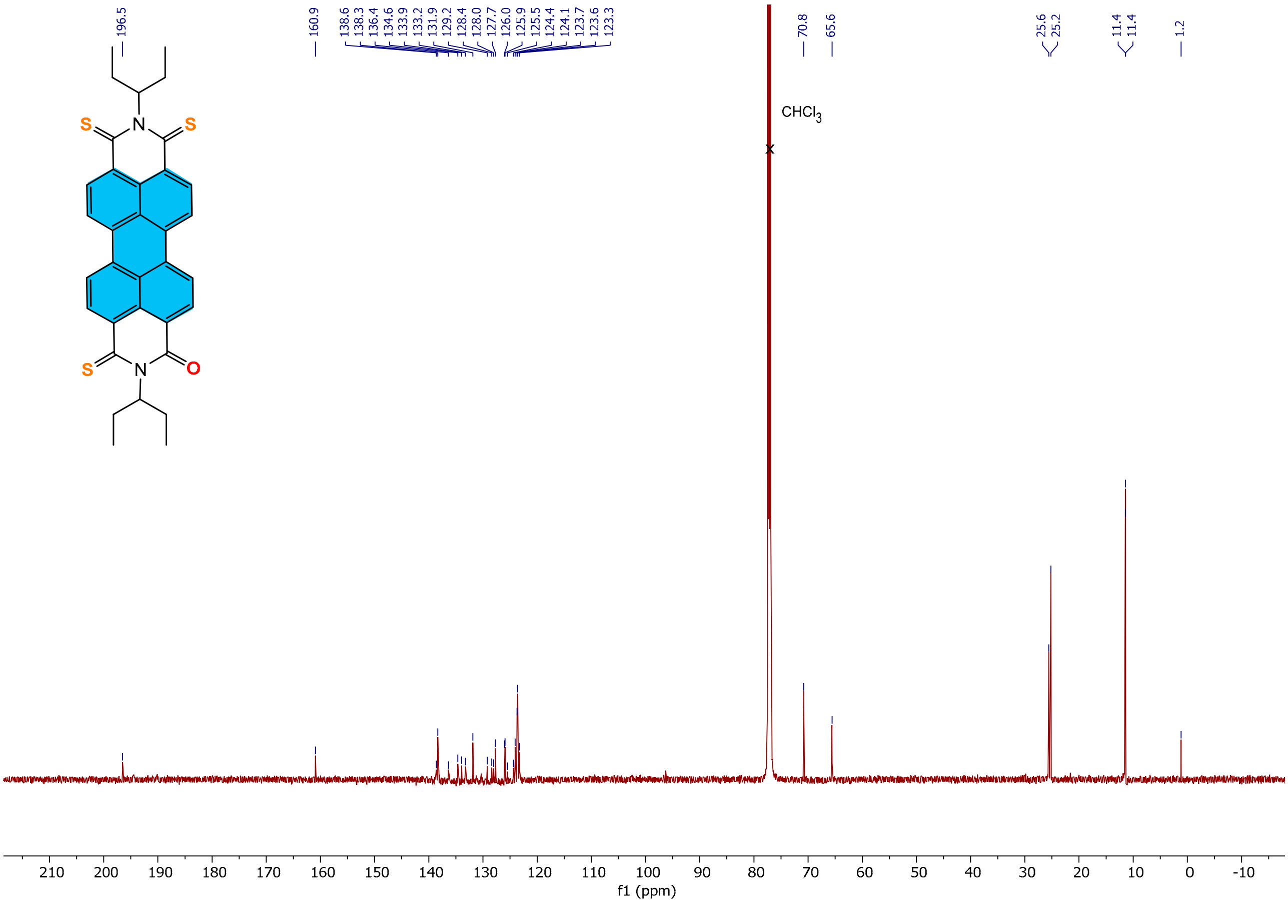

Figure S5. ^{13}C spectrum of **PDI A 3S** recorded in $CDCl_3$

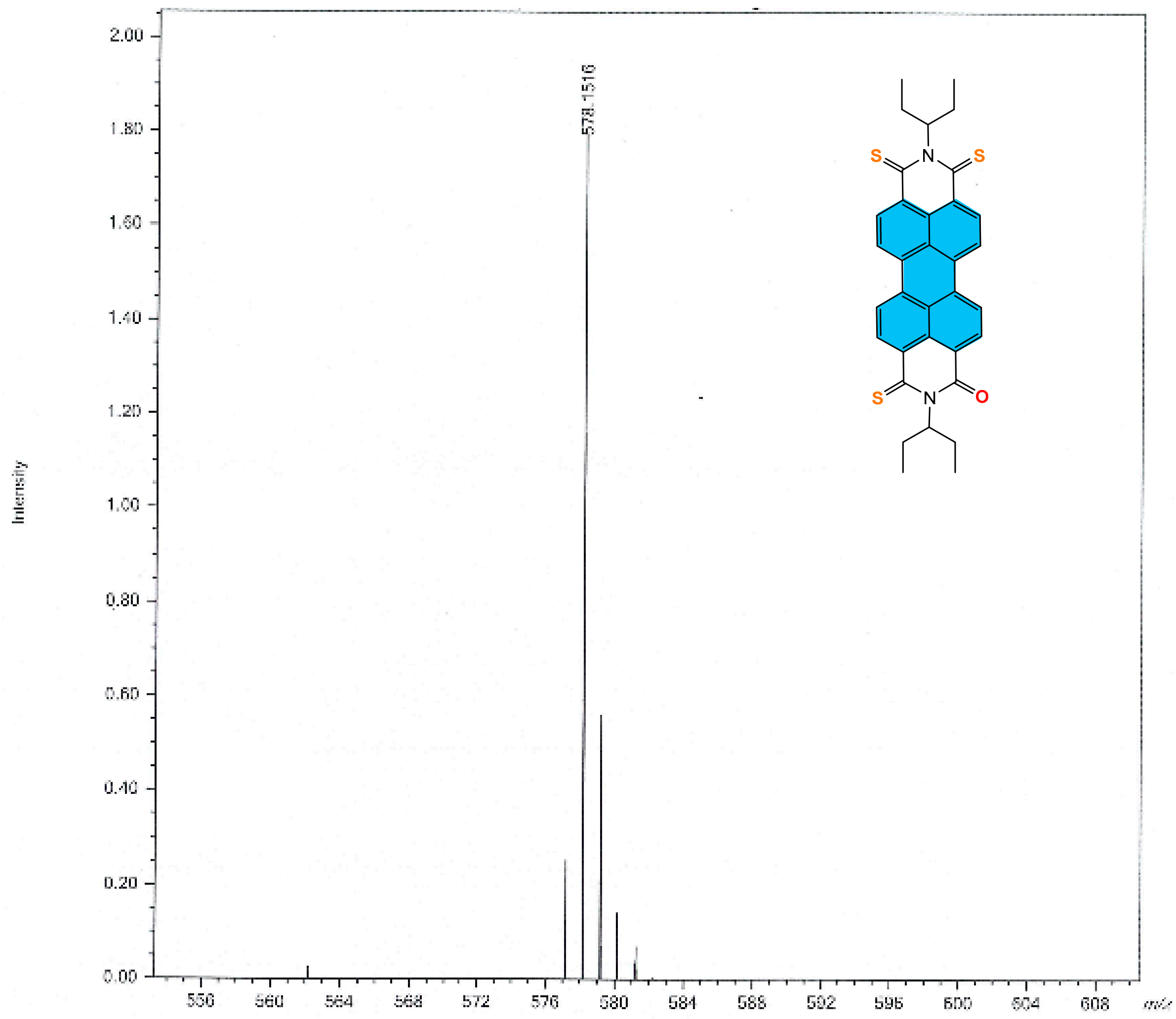

Elemental Composition Estimation

Parameters:

Mass	Tolerance	Electron Mode	Charge	DBE Range	Max Results
578.15156 ± 0.00116	2.0 ppm	Odd	1	-0.5 - 200.0	100

Elements

C 0 - 60 H 0 - 50 O 0 - 2 N 0 - 5 S 0 - 5

Results:

#	Formula	Mass	DBE	Abs. Error (u)	Error (u)	Error (ppm)
1	C34 H30 N2 O S3	578.15257	21.0	0.00102	-0.00102	-1.76

Figure S6. HRMS spectrum of **PDI A 3S**

PDI A 4S:

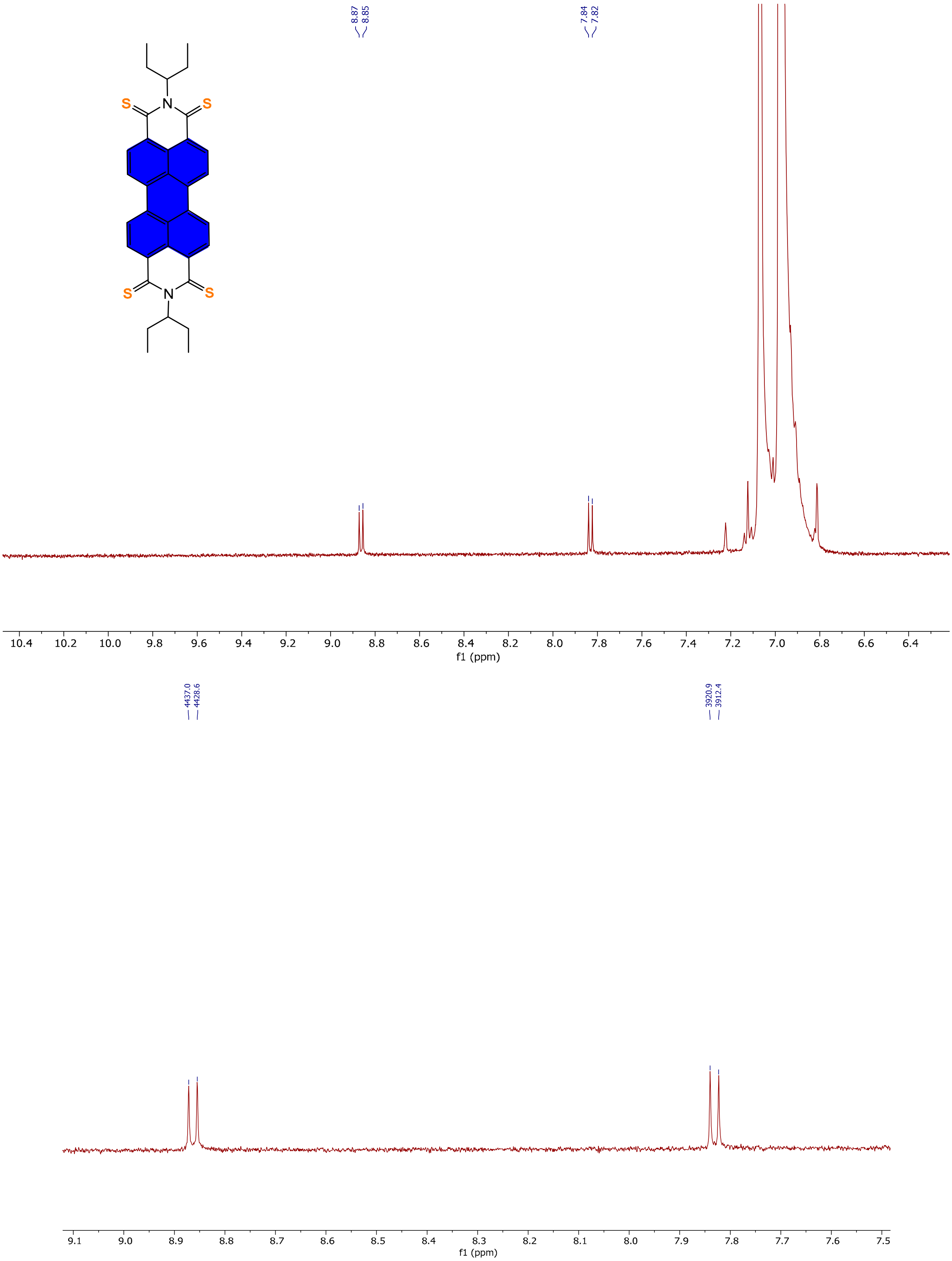

Figure S7. Aromatic part of the ^1H spectrum of **PDI A 4S** recorded in toluene-d8 at 100°C

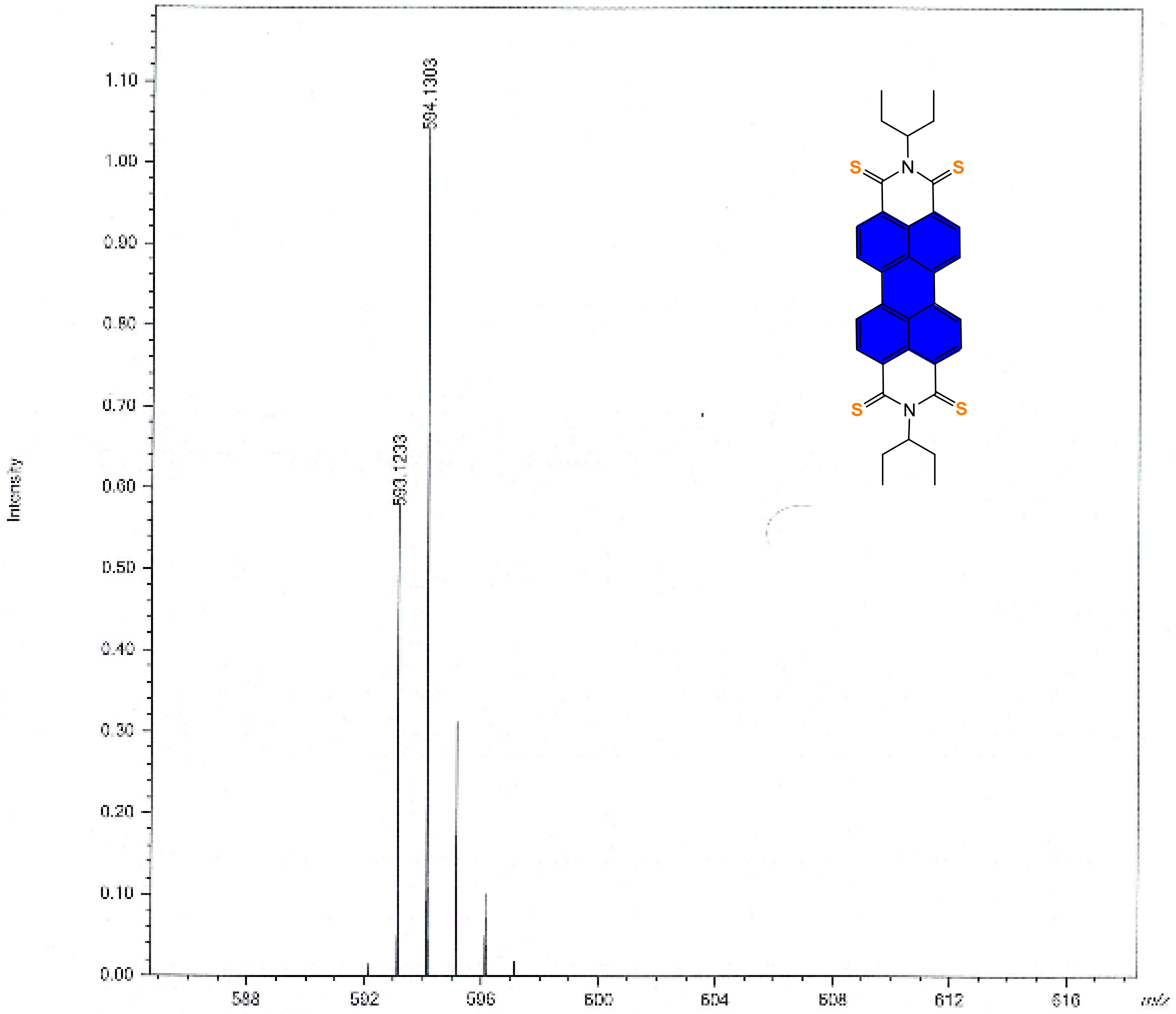

Elemental Composition Estimation

Parameters:

Mass	Tolerance	Electron Mode	Charge	DBE Range	Max Results
594.13033 ± 0.00119	2.0 ppm	Odd	1	-0.5 - 200.0	100

Elements

C	0 - 60	H	0 - 50	O	0 - 0	N	0 - 5	S	0 - 5

Results:

#	Formula	Mass	DBE	Abs. Error (u)	Error (u)	Error (ppm)
1	C34 H30 N2 S4	594.12973	21.0	0.00060	0.00060	1.01

Figure S8. HRMS spectrum of **PDI A 4S**

Compound B:

PDI B 1S:

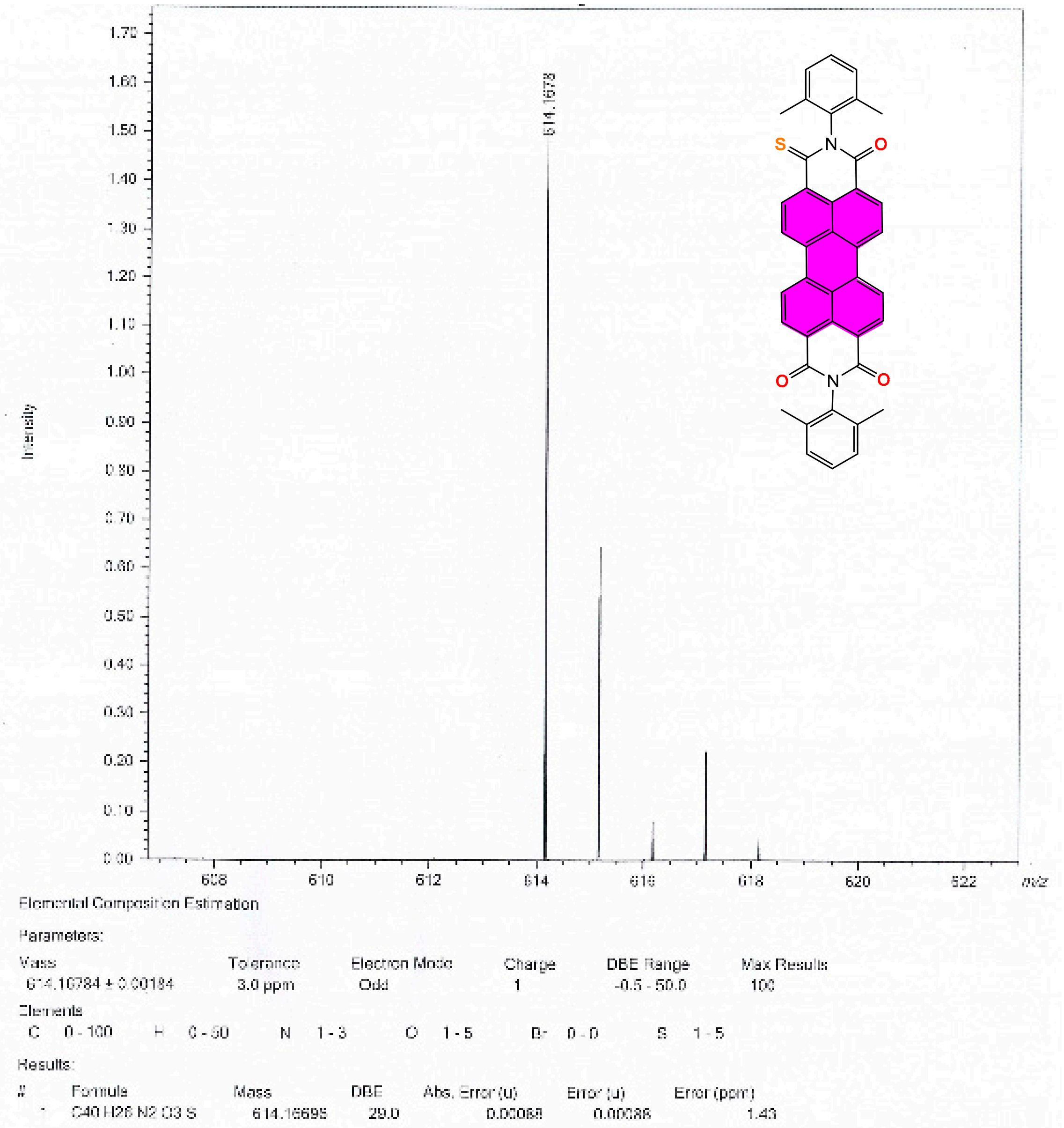

Figure S9. HRMS spectrum of **PDI B 1S**

PDI B 2S-cis:

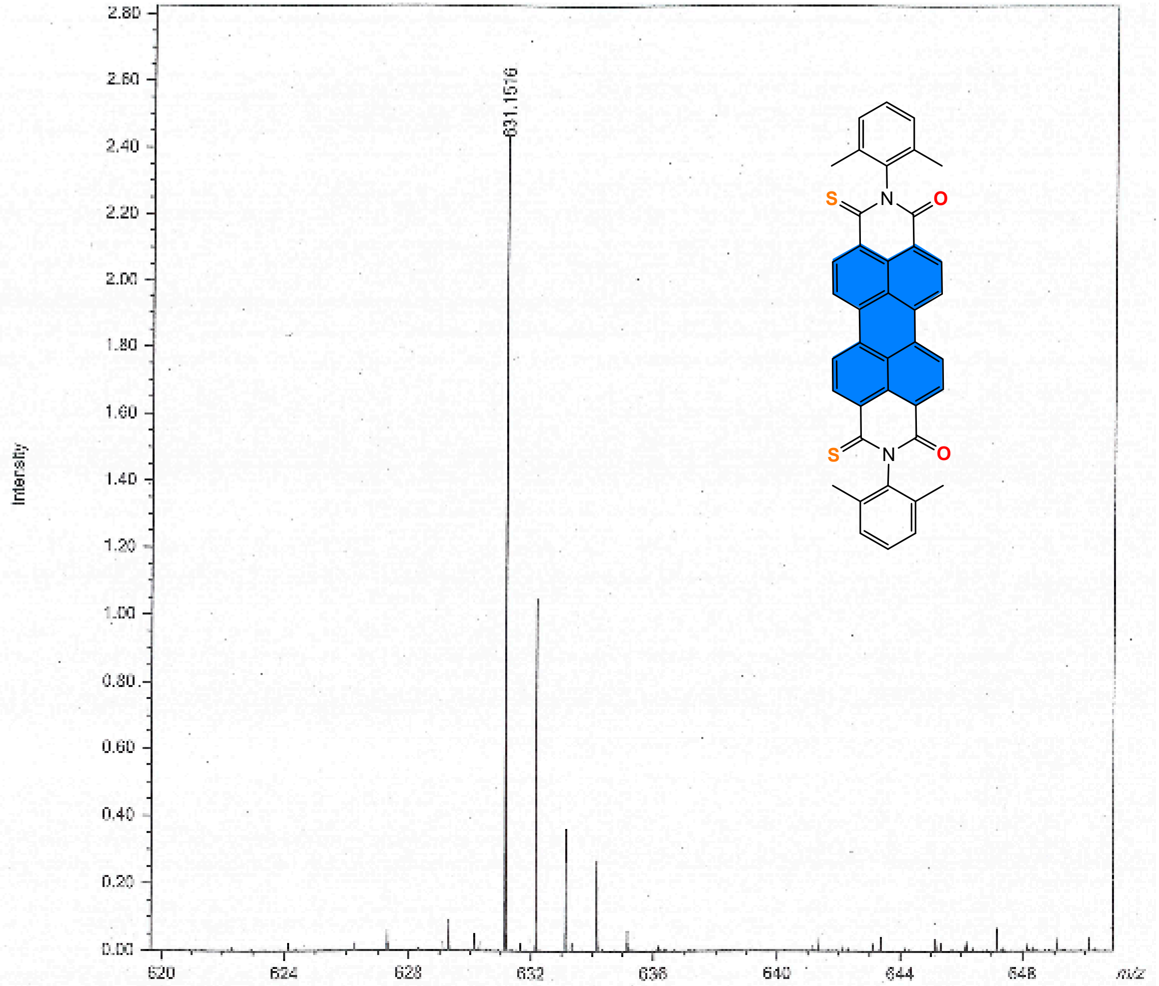

Elemental Composition Estimation

Parameters:

Mass	Tolerance	Electron Mode	Charge	DBE Range	Max Results
631.15157 ± 0.00126	2.0 ppm	Even	+1	-0.5 - 30.0	100

Elements

C 0 - 100 H 0 - 100 N 0 - 5 O 0 - 5 F 0 - 0 S 0 - 4

Results:

#	Formula	Mass	DBE	Abs. Error (u)	Error (u)	Error (ppm)
1	C40 H27 N2 O2 S2	631.15085	28.5	0.00072	0.00072	1.14

Figure S10. HRMS spectrum of **PDI B 2S-cis**

PDI B 2S-trans:

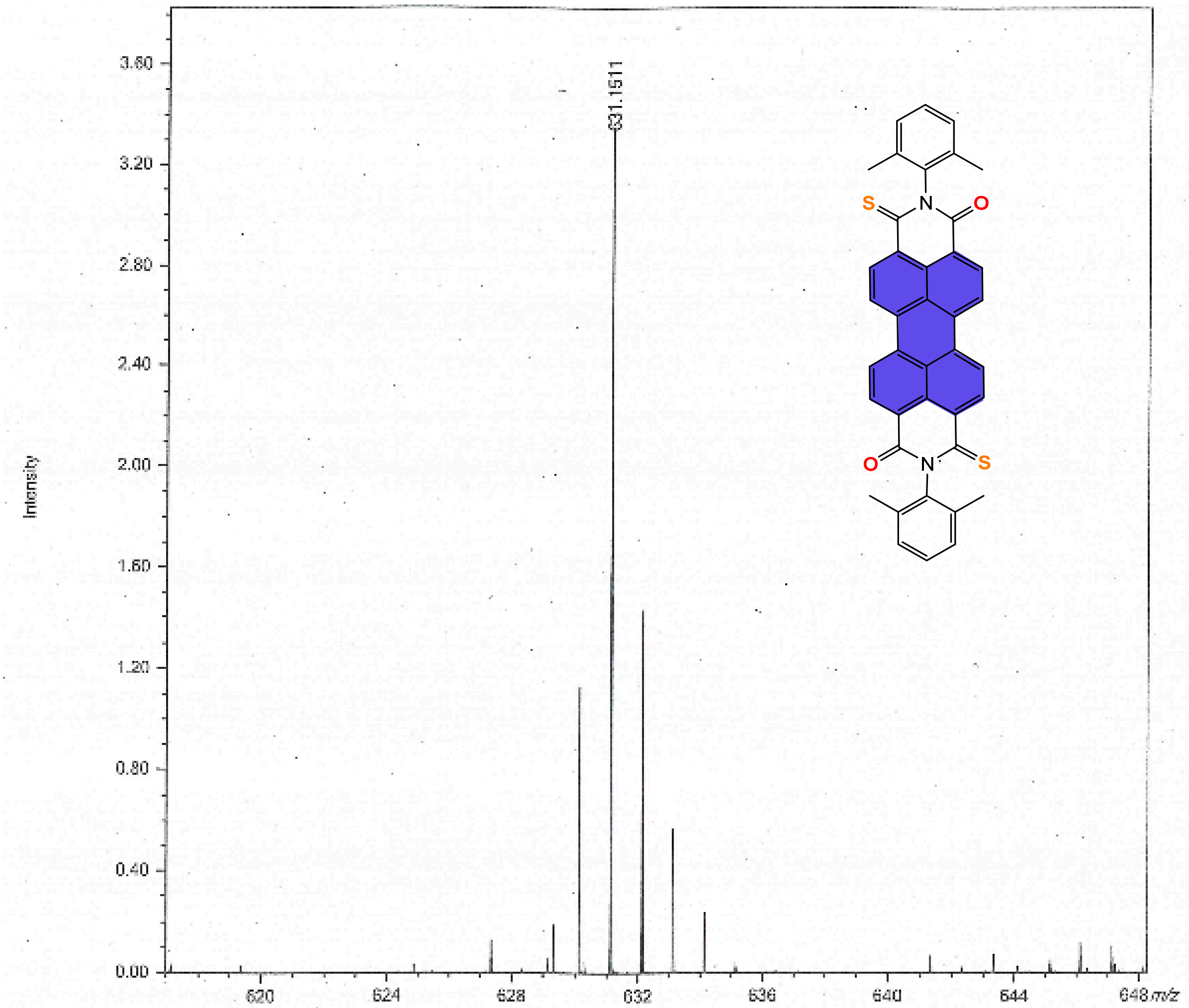

Elemental Composition Estimation

Parameters:

Mass	Tolerance	Electron Mode	Charge	DBE Range	Max Results
631.15107 ± 0.00063	1.0 ppm	Even	+1	-0.5 - 30.0	100

Elements

C 0 - 100	H 0 - 100	N 0 - 5	O 0 - 5	F 0 - 0	S 0 - 4

Results:

#	Formula	Mass	DBE	Abs. Error (u)	Error (u)	Error (ppm)
1	C40 H27 N2 O2 S2	631.15085	28.5	0.00022	0.00022	0.35

Figure S11. HRMS spectrum of **PDI B 2S-trans**

PDI B 3S:

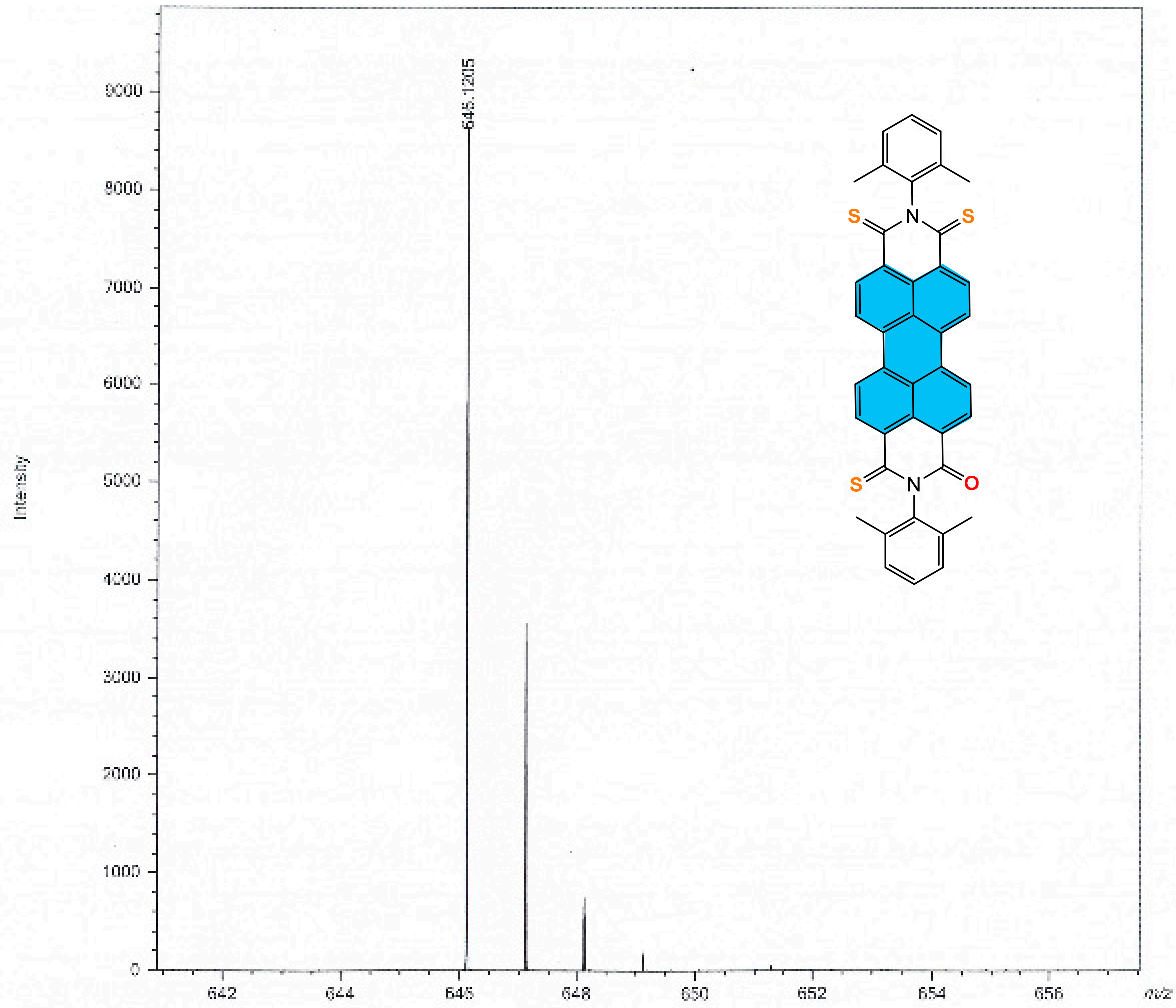

Elemental Composition Estimation

Parameters:

Mass	Tolerance	Electron Mode	Charge	DBE Range	Max Results
646.12051 ± 0.00129	2.0 ppm	Odd	1	-0.5 - 50.0	100

Elements

C 0 - 100 H 0 - 50 N 1 - 3 O 0 - 5 Br 0 - 0 S 1 - 5

Results:

#	Formula	Mass	DBE	Abs. Error (u)	Error (u)	Error (ppm)
1	C40 H25 N2 O S3	646.12127	29.0	0.00076	-0.00076	-1.18

Figure S12. HRMS spectrum of **PDI B 3S**

PDI B 4S:

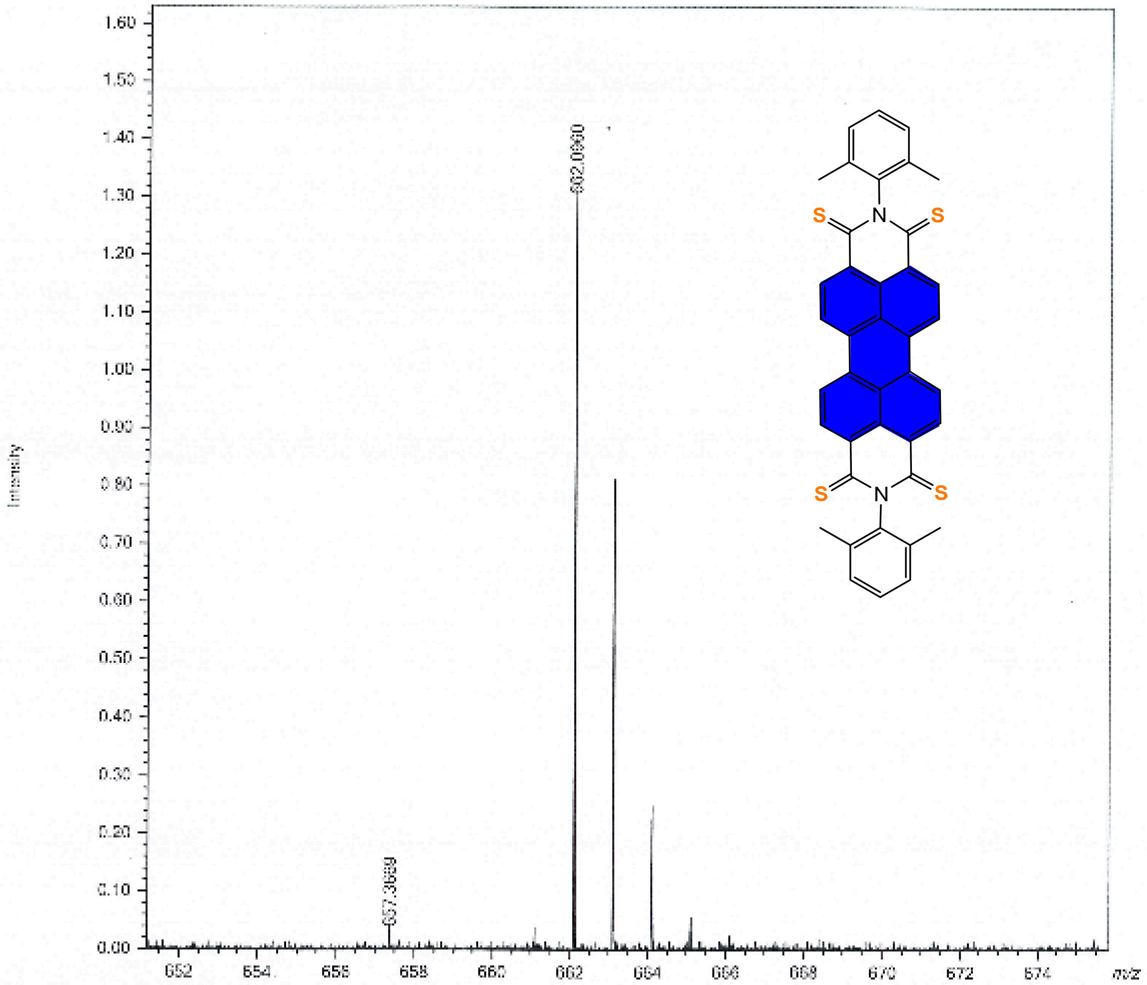

Elemental Composition Estimation

Parameters:

Mass	Tolerance	Electron Mode	Charge	DBE Range	Max Results
662.09595 ± 0.00166	2.5 ppm	Odd	+1	-0.5 - 50.0	100

Elements

C	0 - 100	H	0 - 50	N	1 - 5	O	0 - 0	Br	0 - 0	S	1 - 5

Results:

#	Formula	Mass	DBE	Abs. Error (u)	Error (u)	Error (ppm)
1	C40 H26 N2 S4	662.09733	29.0	0.00138	-0.00138	-2.06

Figure S13. HRMS spectrum of **PDI B 4S**

<u>**Compound C:**</u>

PDI C 4S:

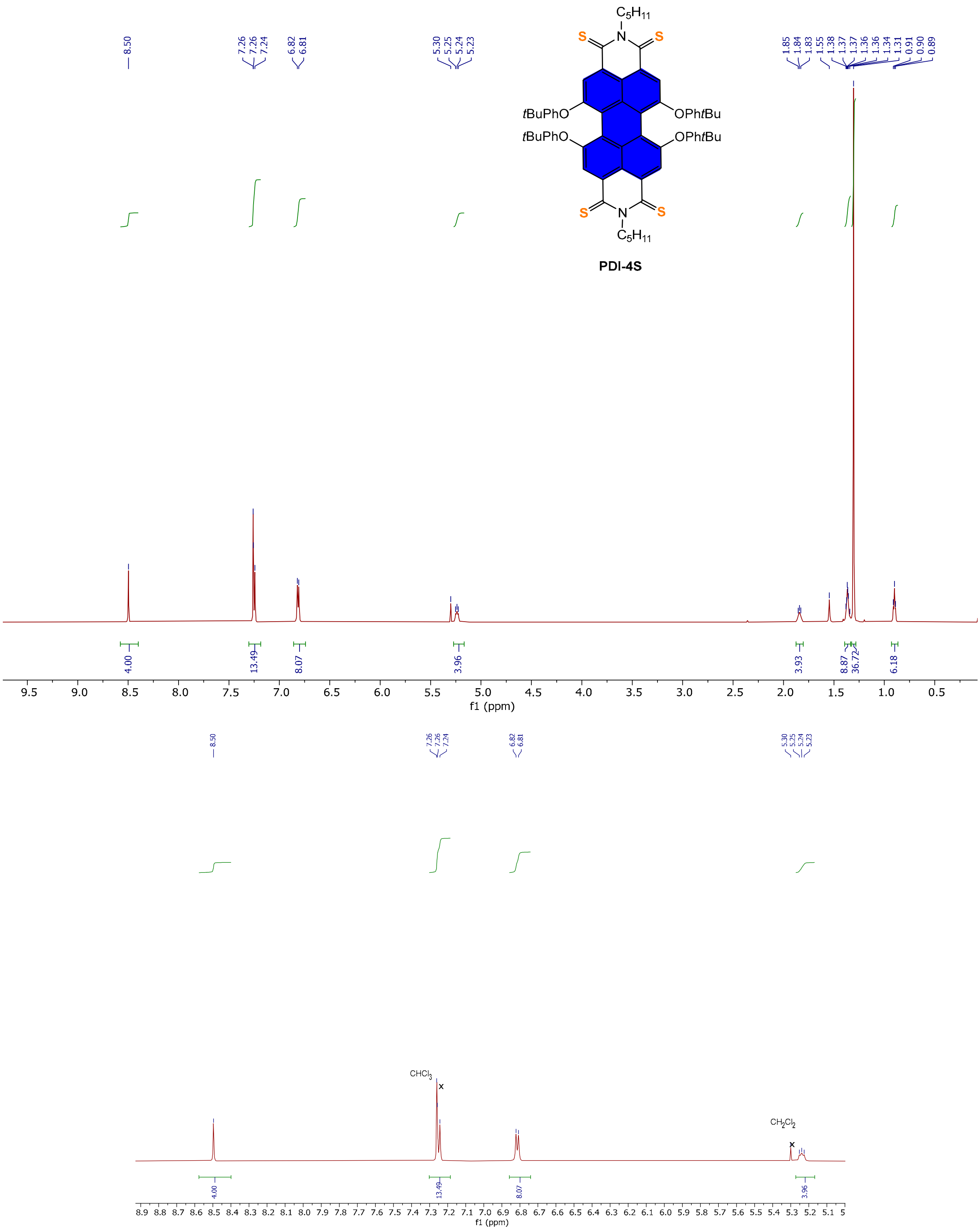

Figure S14. ^{1}H spectrum of **PDI C 4S** recorded in $CDCl_3$ and enlargement of the aromatic part (down)

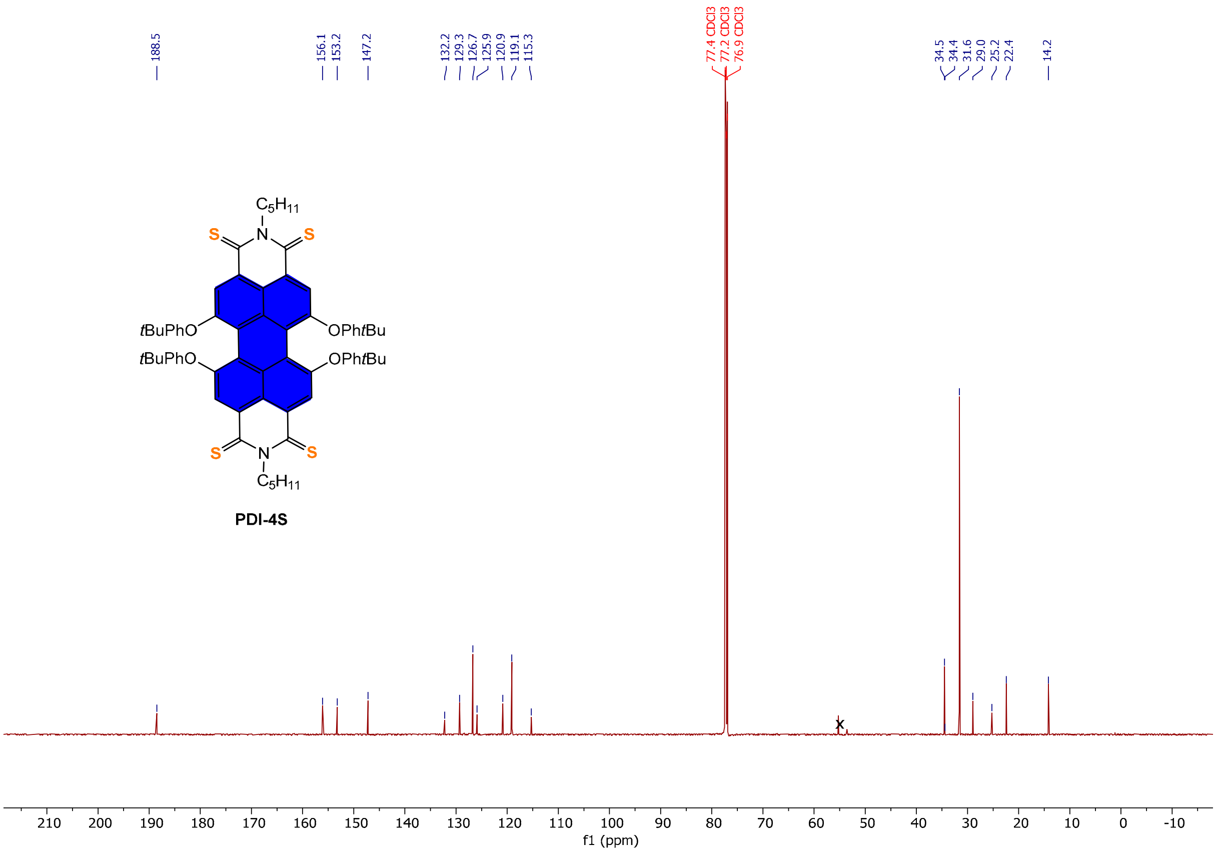

Figure S15. ^{13}C spectrum of **PDI C 4S** recorded in $CDCl_3$

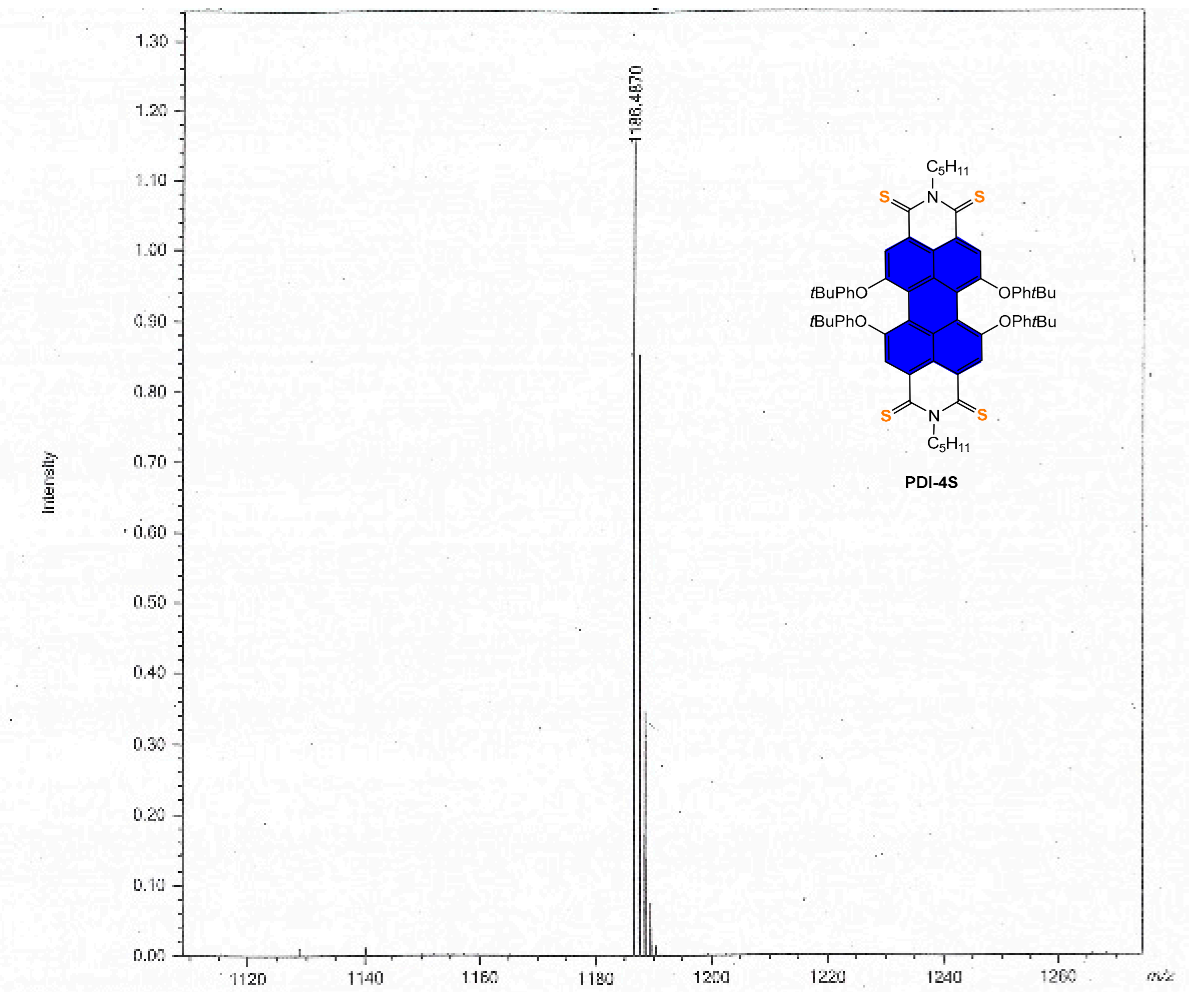

Elemental Composition Estimation

Parameters:

Mass	Tolerance	Electron Mode	Charge	DBE Range	Max Results
1186.48704 ± 0.00237	2.0 ppm	Odd	1	-0.5 - 40.0	100

Elements

C 0 - 100 H 0 - 100 N 0 - 5 O 0 - 5 F 0 - 0 S 0 - 4

Results:

#	Formula	Mass	DBE	Abs. Error (u)	Error (u)	Error (ppm)
1	C74 H78 N2 O4 S4	1186.48499	37.0	0.00205	0.00205	1.73

Figure S16. HRMS spectrum of **PDI C 4S**

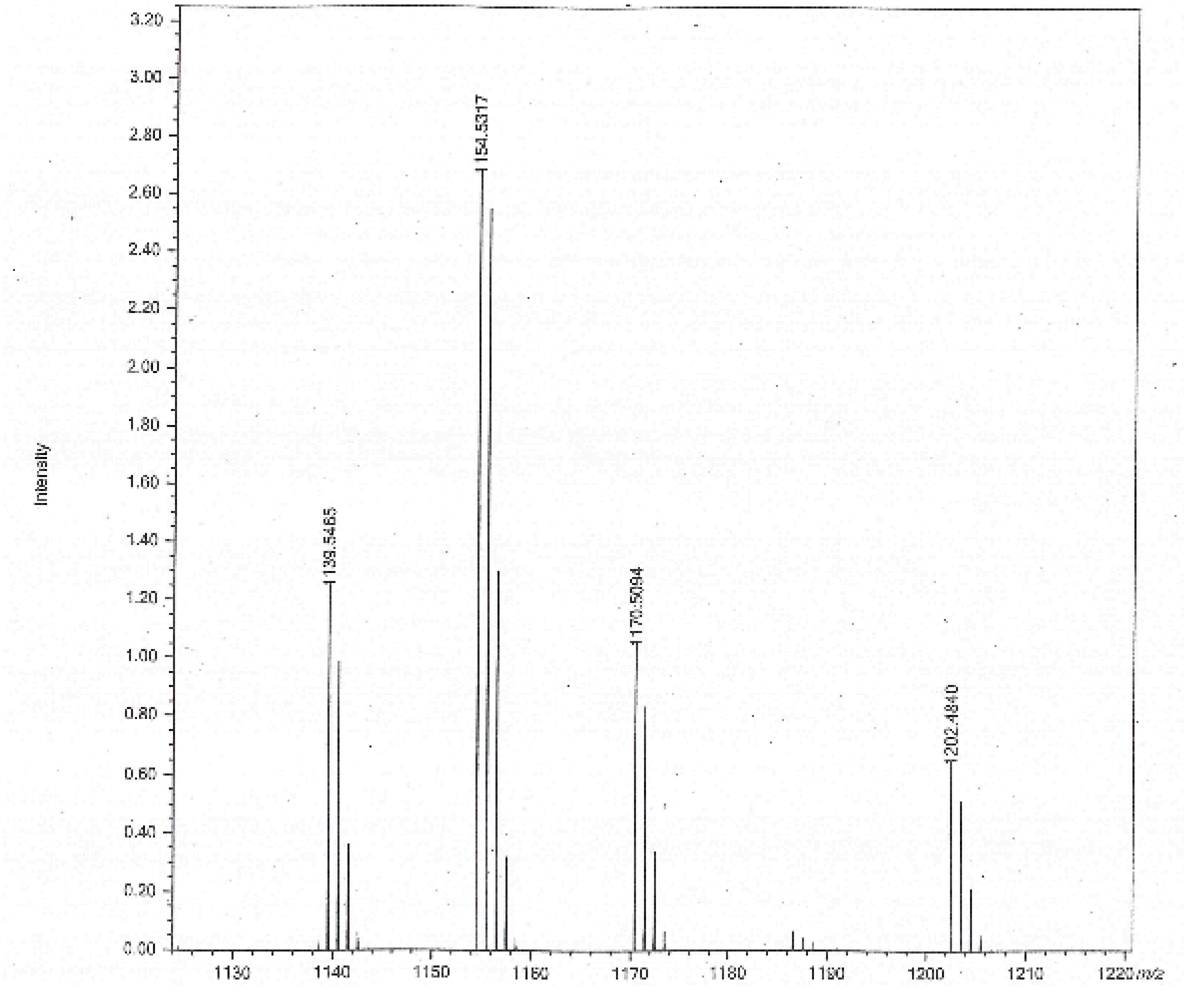

Figure S17. HRMS spectrum of a mixture of **PDI C 1S (m/z = 1138) + : PDI C 2S (m/z = 1154) + PDI C 3S (m/z = 1170)** obtained as traces. The peak at m/z = 1202 could correspond to the PDI-4S derivative on which one phenoxy group was transformed into thiophenoxy group.

Compound D:

PDI D 1S:

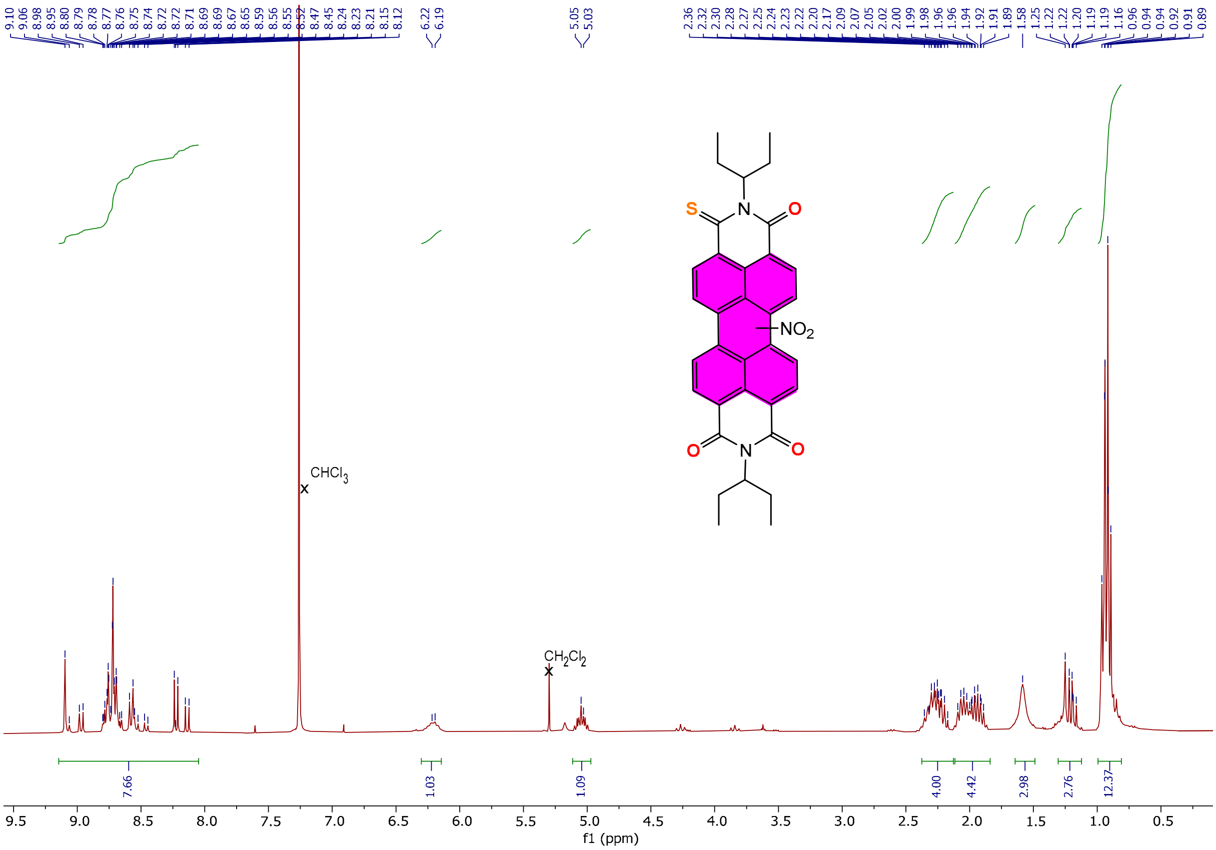

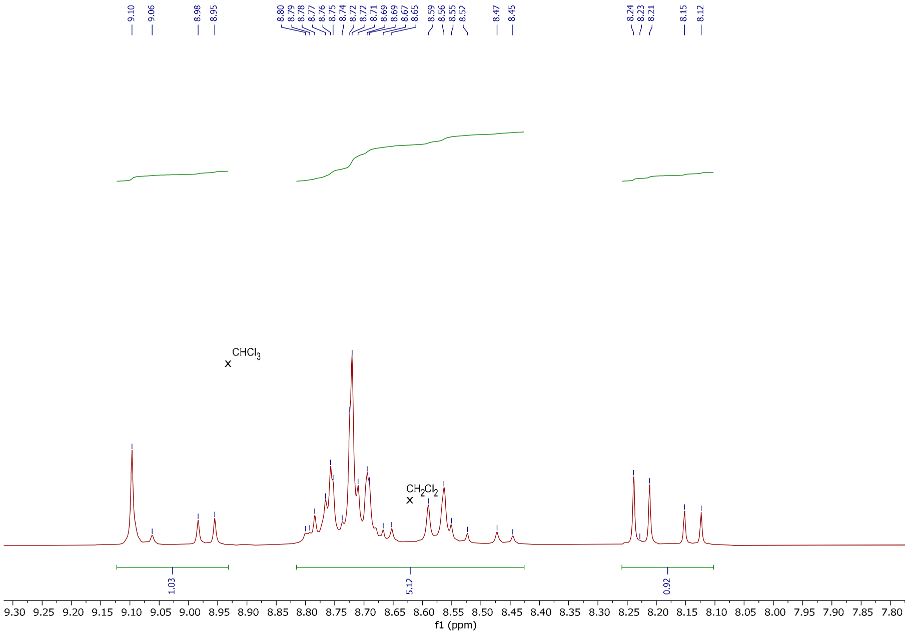

Figure S18. ^{1}H spectrum of **PDI D 1S** recorded in $CDCl_3$ (top) and enlargement of the aromatic part (down)

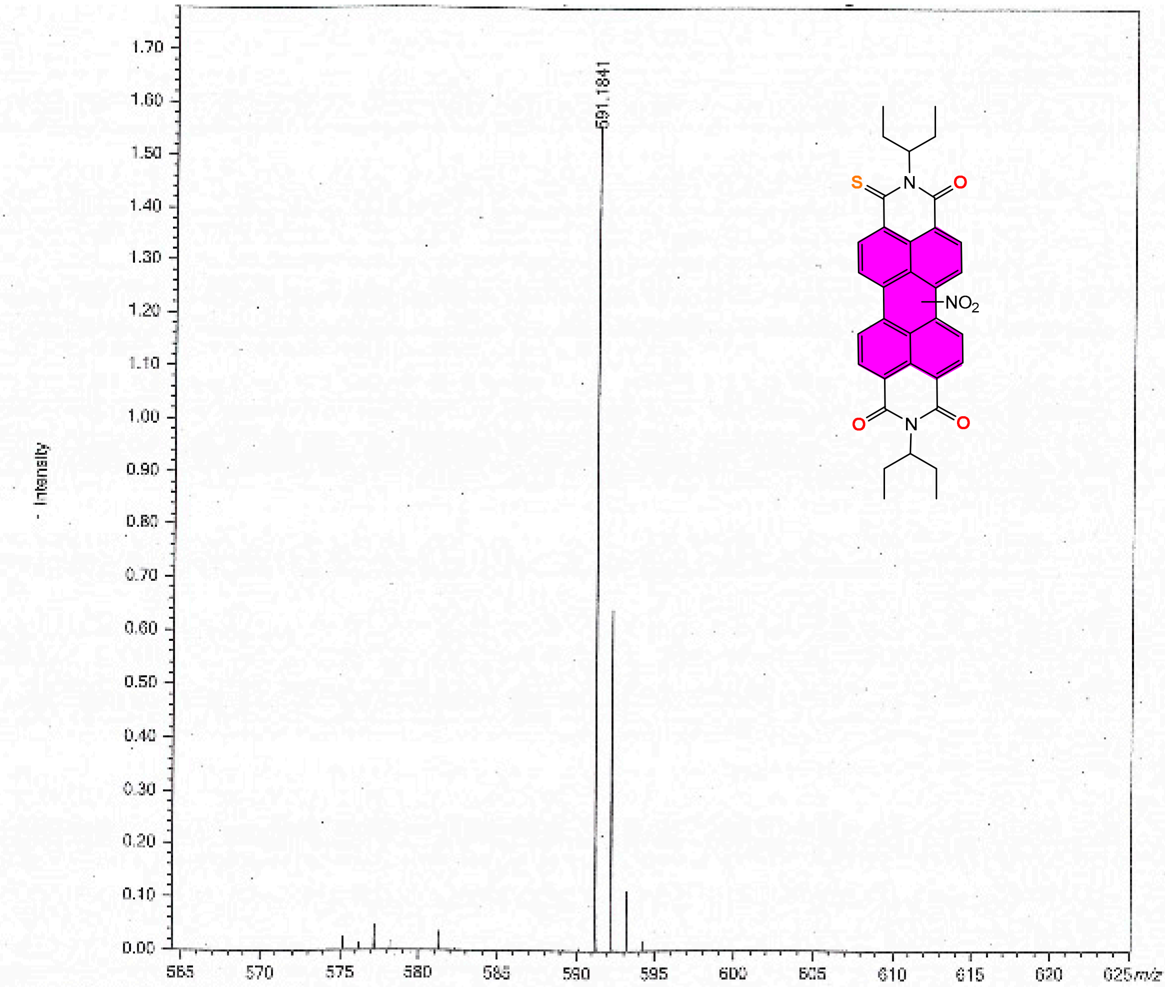

Elemental Composition Estimation

Parameters:

Mass	Tolerance	Electron Mode	Charge	DBE Range	Max Results
591.18413 ± 0.00296	5.0 ppm	Odd	1	-0.5 - 200.0	100

Elements

C 0 - 40 H 0 - 200 N 0 - 5 O 0 - 5 S 0 - 1

Results:

#	Formula	Mass	DBE	Abs. Error (u)	Error (u)	Error (ppm)
1	C34 H29 N3 O5 S	591.18334	22.0	0.00079	0.00079	1.34

Figure S19. HRMS spectrum of **PDI D 1S**

PDI D 2S:

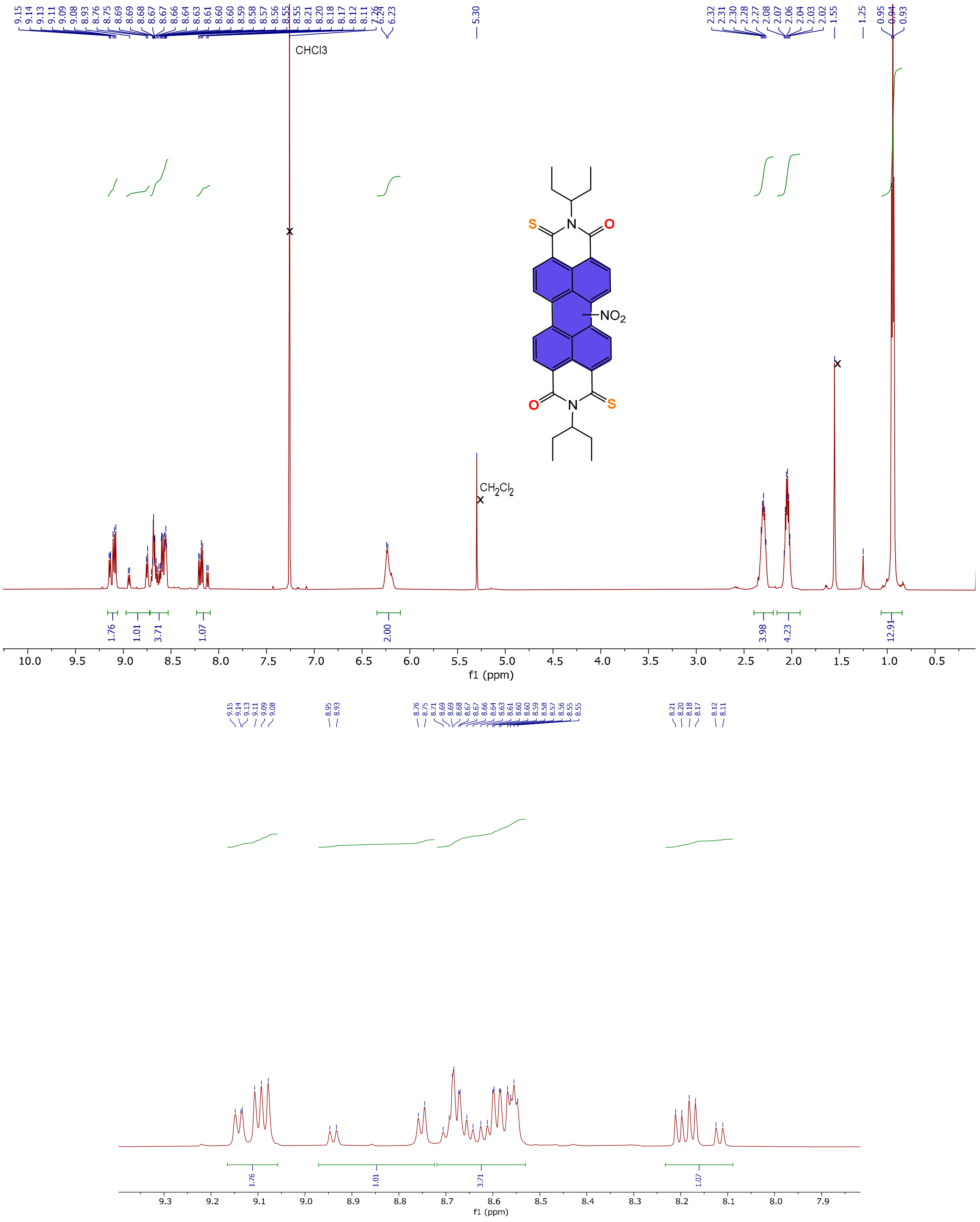

Figure S20. ^{1}H spectrum of **PDI D 2S** recorded in $CDCl_3$ (top) and enlargement of the aromatic part (down)

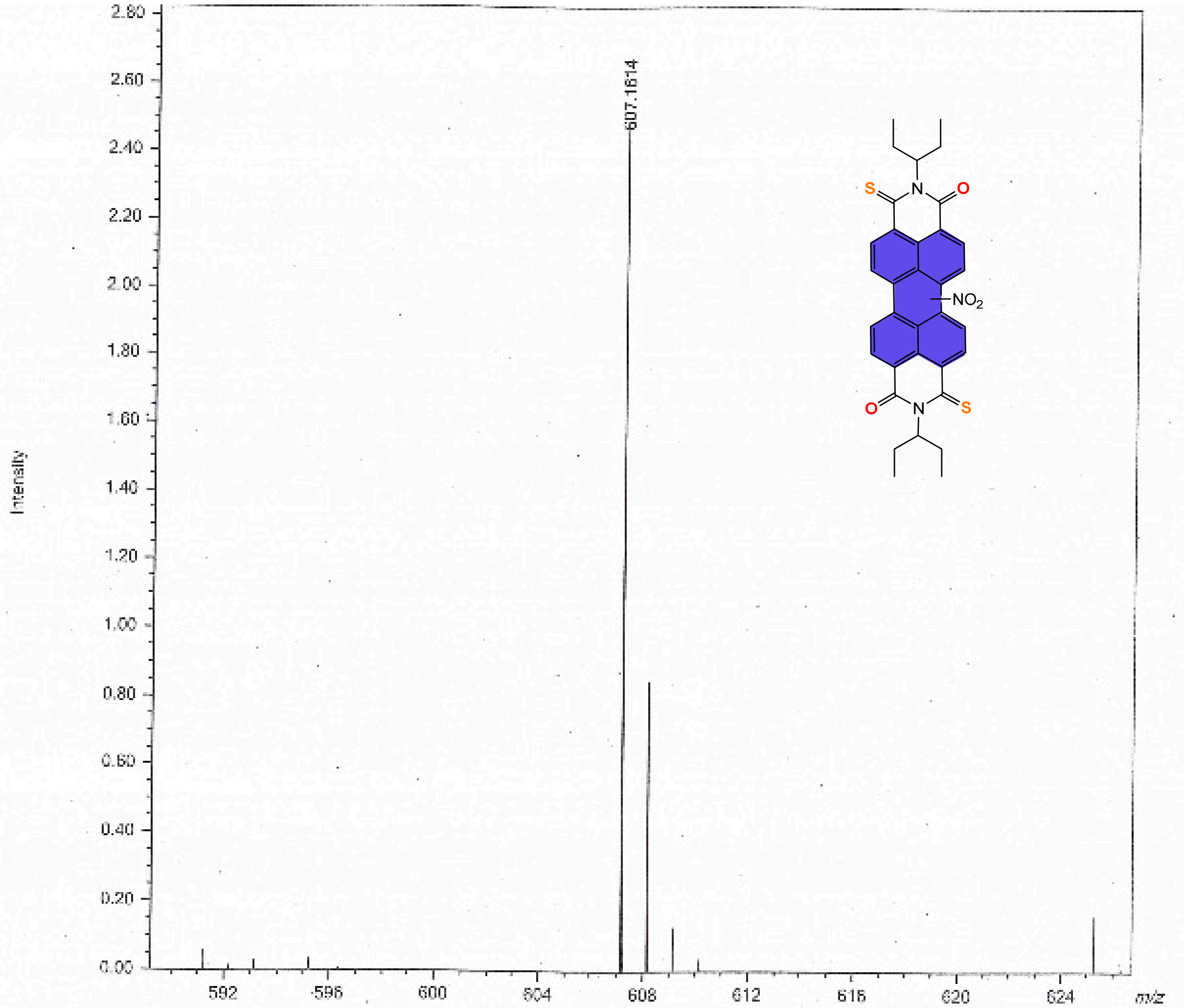

Elemental Composition Estimation

Parameters:

Mass	Tolerance	Electron Mode	Charge	DBE Range	Max Results
607.16140 ± 0.00121	2.0 ppm	Odd	1	-0.5 - 30.0	100

Elements

C 0 - 100 H 0 - 100 N 0 - 5 O 0 - 5 F 0 - 0 S 0 - 4

Results:

#	Formula	Mass	DBE	Abs. Error (u)	Error (u)	Error (ppm)
1	C34 H29 N3 O4 S2	607.16050	22.0	0.00090	0.00090	1.49

Figure S21. HRMS spectrum of **PDI D 2S**

PDI-3S:

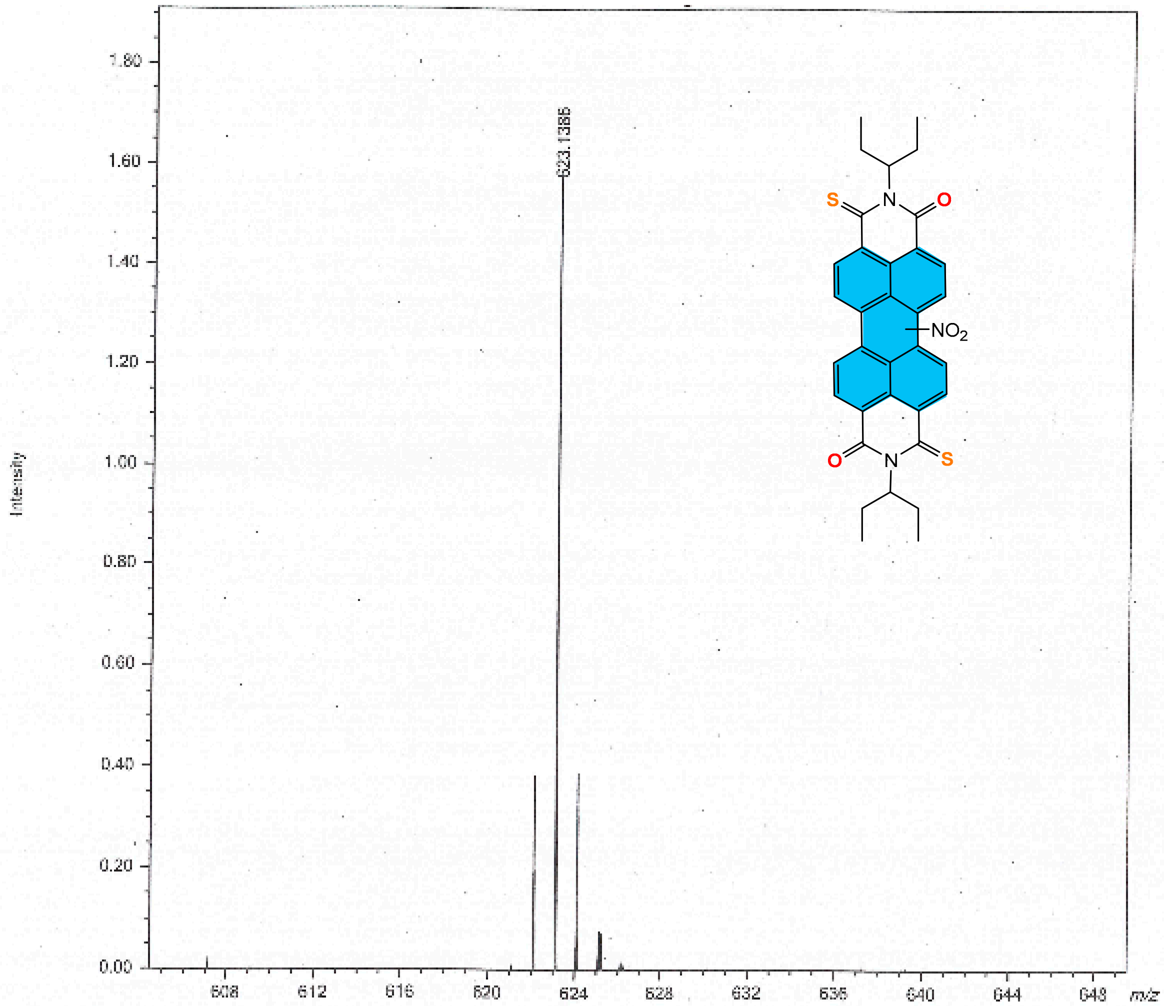

Elemental Composition Estimation

Parameters:

Mass	Tolerance	Electron Mode	Charge	DBE Range	Max Results
623.13863 ± 0.00125	2.0 ppm	Odd	1	-0.5 - 30.0	100

Elements

C 0 - 100 H 0 - 100 N 0 - 5 O 0 - 5 F 0 - 0 S 0 - 4

Results:

#	Formula	Mass	DBE	Abs. Error (u)	Error (u)	Error (ppm)
1	C34 H29 N3 O3 S3	623.13765	22.0	0.00097	0.00097	1.56

Figure S22. HRMS spectrum of **PDI D 3S**

Photochemical degradation study of thionated PDI derivatives:

PDI A:

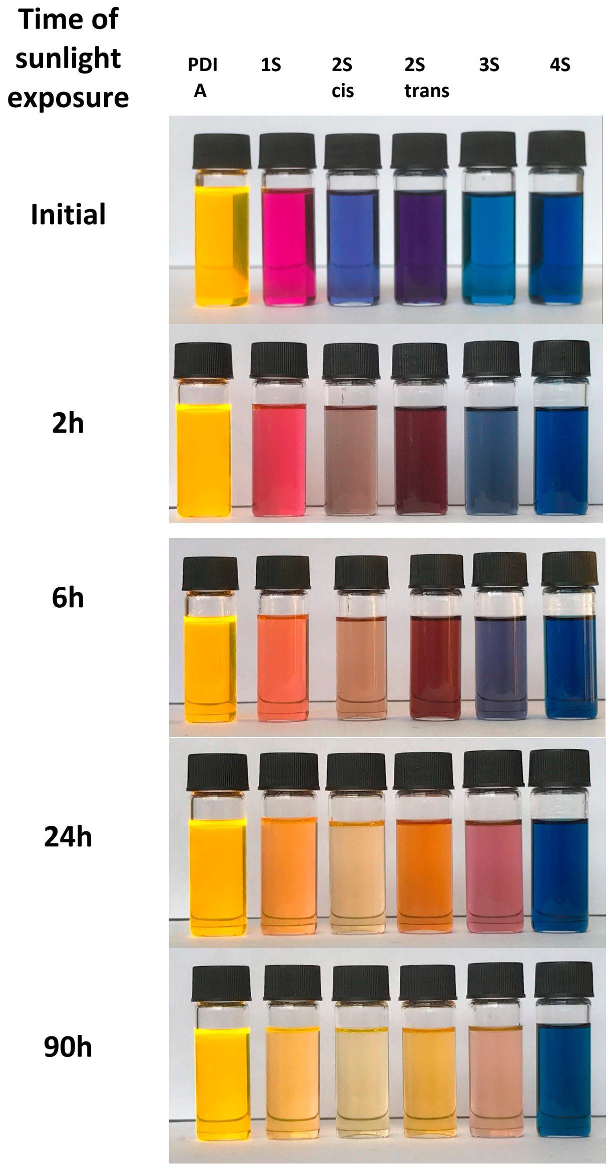

Figure S23. Evolution of photochemical degradation of **PDI A** and its thionated derivatives under sunlight irradiation in oxygen saturated solution in CH_2Cl_2

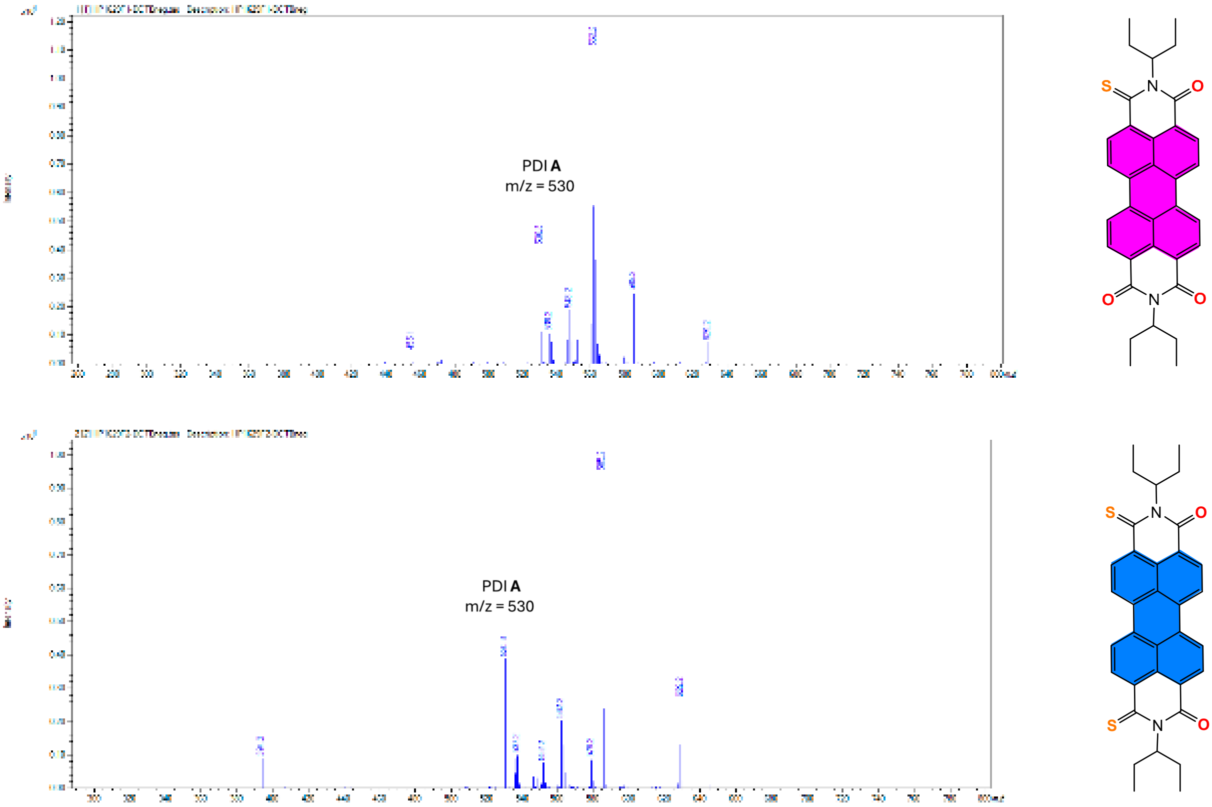

Figure S24. MS spectra of **PDI A 1S** (top) and **PDI A 2S-cis** (down) from solutions in CH_2Cl_2 saturated with oxygen after sunlight irradiation

1. Hendsbee, A. D.; Sun, J.-P.; Law, W. K.; Yan, H.; Hill, I. G.; Spasyuk, D. M.; Welch, G. C., Synthesis, Self-Assembly, and Solar Cell Performance of N-Annulated Perylene Diimide Non-Fullerene Acceptors. *Chem. Mater.* **2016,** *28* (19), 7098–7109.
2. Demmig, S.; Langhals, H., Leichtlösliche, lichtechte Perylen-Fluoreszenzfarbstoffe. *Chem. Ber.* **1988,** *121* (2), 225–230.
3. Perrin, L.; Hudhomme, P., Synthesis, Electrochemical and Optical Absorption Properties of New Perylene-3,4:9,10-bis(dicarboximide) and Perylene-3,4:9,10-bis(benzimidazole) Derivatives. *European Journal of Organic Chemistry* **2011,** *2011* (28), 5427-5440.
4. Lee, Y.-L.; Chou, Y.-T.; Su, B.-K.; Wu, C.-c.; Wang, C.-H.; Chang, K.-H.; Ho, J.-a. A.; Chou, P.-T., Comprehensive Thione-Derived Perylene Diimides and Their Bio-Conjugation for Simultaneous Imaging, Tracking, and Targeted Photodynamic Therapy. *Journal of the American Chemical Society* **2022,** *144* (37), 17249-17260.
